# Cultivated Grapevines Represent a Symptomless Reservoir for the Transmission of Hop Stunt Viroid to Hop Crops: 15 Years of Evolutionary Analysis

**DOI:** 10.1371/journal.pone.0008386

**Published:** 2009-12-24

**Authors:** Yoko Kawaguchi-Ito, Shi-Fang Li, Masaya Tagawa, Hiroyuki Araki, Masafumi Goshono, Shingen Yamamoto, Mayumi Tanaka, Masako Narita, Kazuaki Tanaka, Sheng-Xue Liu, Eishiro Shikata, Teruo Sano

**Affiliations:** 1 United Graduate School of Agricultural Sciences, Iwate University, Morioka, Japan; 2 State Key Laboratory of Biology of Plant Diseases and Insect Pests, Institute of Plant Protection, Chinese Academy of Agricultural Sciences, Beijing, People's Republic of China; 3 Faculty of Agriculture and Life Science, Hirosaki University, Hirosaki, Japan; 4 Key Oasis Eco-agriculture Laboratory of Xinjiang Production and Construction Group, Shihezi, People's Republic of China; 5 Graduate School of Agriculture, Japan Academy, Hokkaido University, Sapporo, Japan; Institute of Molecular and Cell Biology, Singapore

## Abstract

Hop stunt was a mysterious disorder that first emerged in the 1940s in commercial hops in Japan. To investigate the origin of this disorder, we infected hops with natural Hop stunt viroid (HpSVd) isolates derived from four host species (hop, grapevine, plum and citrus), which except for hop represent possible sources of the ancestral viroid. These plants were maintained for 15 years, then analyzed the HpSVd variants present. Here we show that the variant originally found in cultivated grapevines gave rise to various combinations of mutations at positions 25, 26, 54, 193, and 281. However, upon prolonged infection, these variants underwent convergent evolution resulting in a limited number of adapted mutants. Some of them showed nucleotide sequences identical to those currently responsible for hop stunt epidemics in commercial hops in Japan, China, and the United States. Therefore, these results indicate that we have successfully reproduced the original process by which a natural HpSVd variant naturally introduced into cultivated hops was able to mutate into the HpSVd variants that are currently present in commercial hops. Furthermore, and importantly, we have identified cultivated grapevines as a symptomless reservoir in which HSVd can evolve and be transmitted to hop crops to cause epidemics.

## Introduction

Viroids are the smallest known pathogens and cause severe to mild diseases in economically important crop species [1]. They are single-stranded, circular, and self-replicating non-coding RNAs, with the size ranging ∼250–400 nucleotides. Viroid replication is dependent on host transcriptional machinery, and pathogenicity depends entirely on interactions with cellular components of the host [2]–[7]. The small size and unique molecular structure of viroid genomes makes them an attractive system to analyze molecular features responsible for pathogenesis [8]–[14], RNA transport [15]–[18], or molecular evolution and adaptation to specific host species [19]–[26].

Abnormally dwarfed hop crops were first recognized in the 1940s in Japan, and subsequently became epidemics over the next two decades [27]. The diseased hops were called ‘dwarf hop’ or ‘cedar-shaped hop’ based on the reduction in their plant height by up to 50% compared with healthy plants [28]. Additionally, the disease hops only contained one-third to one-half the amount of alpha-acids contained in healthy hops, which decreased their suitability for beer production [28]. The causal agent was eventually identified to be a small infectious RNA pathogen, and was named *Hop stunt viroid* (HpSVd) [29]. For many years this viroid was restricted to Japan, with the exception being rhizomes that were introduced from Japan into South Korea [30]. However, in 2004, the disease was observed for the first time in the state of Washington in the US, the world's second-largest hop-producing country [31]. More recently, in 2007, HpSVd was detected in the Xinjiang autonomous region of China, where ∼5000 metric tons of hops are produced annually [32].

Hop is a perennial herb that originated in a region between the Mediterranean Sea and the Caucasus. Cultivation began in the mid-8th century in Germany and was later developed in several European countries, eventually being introduced to Japan in the late 19th century. In Japan, a full-scale breeding program was started from the selection and/or crossing of foreign varieties, resulting in the propagation of foreign hop varieties, as well as the viruses and viroids associated with them [28], [33]. Therefore, importation of contaminated mother stocks had the potential to spread viruses and viroids through diseased material that was clonally propagated either by rooted cuttings or by rhizomes. For HpSVd, however, this was not the case. HpSVd emerged for the first time in Japan ∼70 years ago, which was 30–40 years after full-scale cultivation had been started. HpSVd had also never been reported in any other hop-growing area of the world until recently.

Soon after its discovery, HpSVd was found to have infected grapevines and fruit trees [34]–[36]. Namely, the majority of citrus varieties [37] and stone fruits [38]–[41], including plum, peach, apricot, almond and Jujube, were shown to harbor the viroid latently. However, special variants have been shown to cause cachexia disease in citrus [42] and dapple fruit in peaches and plums [43]. HpSVd is now considered to be a ubiquitous, and genetically variable, pathogen that has spread among the varieties of fruit trees cultivated worldwide.

The aim of this research was to analyze how a series of natural HpSVd variants, which had adapted to various host crops, could incite disease symptoms in hop crops, and how their nucleotide sequence was affected following introduction into this sensitive host species. The results were anticipated to identify which of the natural HpSVd variants was the origin for hop stunt epidemics in Japan, and to shed light on the molecular process by which viroid fix mutations when adapting to new host species. For these studies, we artificially infected hops with four major HpSVd variants associated with current epidemics and established a system in which to analyze the ability of the hop host to adapt to these viroids over a long-term, persistent infection. Here, we analyze data from 15 years of persistent infection to evaluate pathological and evolutionary characteristics of four HpSVd variants shown to cause mild to severe hop stunt disease. In parallel, a major variant of HpSVd-grapevine was introduced into hops and was shown to be modified into natural HpSVd-hop variants currently endemic in commercial hops. Based on this analysis, we discuss the origin of hop stunt epidemics.

## Results

### Variations in Natural HpSVd-Hop and HpSVd-Grapevine Isolates

Prior to presenting the data obtained from our experiments, the diversity of nucleotide sequences identified for HpSVd isolates associated with current epidemics of commercial hops and cultivated grapevines are detailed.

#### Natural HpSVd-hop in Japan

**Figure 1:**
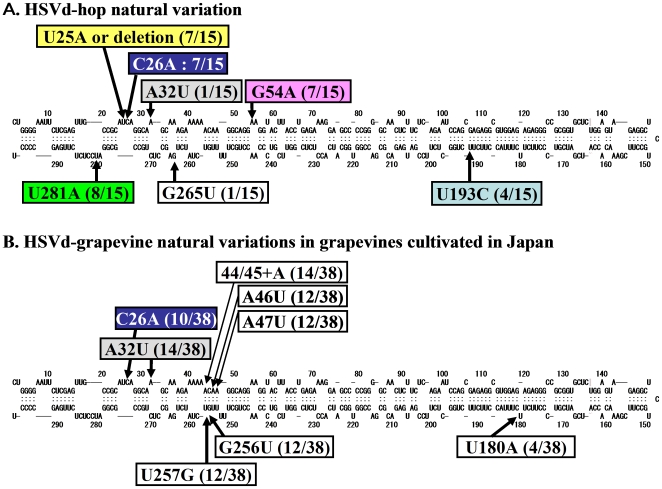
A schematic representation of the major sequence variations in natural HpSVd isolates from hop and grapevine. Variations found in natural HpSVd-hop isolates in the commercial hops in Japan (A) and in natural HpSVd-grapevine isolates from grapevines cultivated in Japan (B). The secondary structure model was produced by M-fold using the sequence in the inoculum; for example, hJType variant for the natural HpSVd-hop. Mutations and their location in the predicted secondary structure model are shown in boxes, and the number of mutants/total numbers of cDNA sequenced between parenthesis.

Seven major natural HpSVd-hop variants were previously reported in commercial hops in Northern Japan [44]–[46]. In this study, 18 additional cDNA clones, six per isolate, were randomly selected from three natural HpSVd-hop isolates collected in 2004 from Northern Japan. Sequence analysis of these latter isolates identified two major variants, designated hKY04-1 and 04-7. These variants were aligned with the seven known variants previously published and hKY04-1 was identified as a new hop variant, although it was identical to a variant associated with cultivated grapevines. Variant 04-7 was identical to hAIw36, one of the seven previously reported hop variants. Consequently, we have identified a total of eight major natural HpSVd variants from commercial hops in Japan: hJType, hKY04-1, hAIw36, hKIw, hAIw, hKF76, hKFKi, and hSIw. The majority of point mutations present in these variants were associated with nucleotide positions: 25, 26, 32, 54, 193, 265 and 281 (Fig. 1A). Combinations of nucleotides at each of these seven positions in the eight major variants are summarized in Table 1. Of note, changes at positions 32 and 265 were only found in the hSIw isolate with a lower frequency. Since HpSVd is variable in length (294–303 nts), the numbering of the nucleotides hereafter is based on the numbering used in the HpSVd-hop sequence (hJType: accession E00276).

**Table 1:** Nine major HpSVd variants in the commercial hop populations.

			Combination of Seven Nucleotides						
	Nucleotide No.		25* ^1^	26	32	54	193	265	281
		Location							
HpSVd-grapevine* ^2^		world wide	U	C	A	G	U	G	U
HpSVd-hop Types									
I	hKY04-1	Japan	U	C	A	G	U	G	U
II	hAIw36	Japan	-* ^3^	C	A	G	U	G	U
III	hJType	Japan	U	C	A	A	U	G	U
IV	hKIw	Japan	U	C	A	G	C	G	A
V	hAIw	Japan	U	A	A	G	C	G	A
VI	hKF76	Japan	A	A	A	A	U	G	A
VII	hKFKi	Japan	A	A	A	A	C	G	A
	hChina07-2-3	China	A	A	A	A	C	G	A
VIII	hSIw	Japan	C	U	U	A	C	U	A
IX	hChina07-2-4	China	U	A	A	A	C	G	U

*1. Nucleotide numbering refers to numbering used in the HpSVd–hop sequence, hJType (Accession no. E00276).

*2. Nucleotide sequence of HpSVd-grapevine provided for reference.

*3. (-) means deletion.

#### Natural HpSVd-hop in China

Five natural HSVd-hop isolates were collected from Xinjiang, China, and 2–7 replicates from each isolate were sequenced. A total of 23 independent cDNA clones were identified and aligned with the eight major variants identified from Japan. One major variant, designated hChina07-2-4 in Table 1, contained three of the seven changes found in the Japanese isolates at positions 26, 54, and 193. However, since the combination of changes was unique, it was identified as a new type of variant, IX. Similarly, another major variant, hChina07-2-3, contained five of the seven Japanese variations at positions 25, 26, 54, 193 and 281, and was identical to hKFKi. Three other minor variants, however, contained additional unique changes at positions 49, 257, or 281. For example, hChina07-18-1, included variations at 26, 54, and 193, which were consistent with the Japanese isolates, although variations present at 49 and 257 were unique to the Chinese isolates.

#### Natural HpSVd in cultivated grapevines

A total of 113 grapevine samples were collected in Japan, most of them from different cultivars. Based on Northern hybridization and/or RT-PCR assays, 73 were positive for HpSVd. Full-length HpSVd cDNAs were amplified from 24 different cultivars for cloning, and five to ten cDNA clones per sample were then sequenced. A majority of the samples only contained one major HpSVd variant, but some contained multiple sequence variants. A total of 38 major HpSVd variants were identified and aligned to detect natural sequence changes associated with grapevines cultivated in Japan (Supp. Table S1): major variations were present at positions 26, 32, 44/45, 46, 47, 180, 256 and 257 (Fig. 1B). Phylogenetic analysis showed that HpSVd-grapevine variants were divided into two clusters (Supp. Fig. S1), with one cluster deriving from the original HpSVd reported from grapevine [47], and the second from the grapevine cultivar, “Riesling” [48]. The former group appeared to be more abundant in the natural population and all changes, except at positions 44/45 and 180, were different between the two groups.

Since grapevines are cultivated throughout the world and commonly harbor HpSVd, sequence diversity of the HpSVd-grapevine isolates identified were compared against variants deposited in the subviral RNA database (SubViral RNA data base, [49]). Of the 62 sequence records available from the database, 18 were deposited at least twice. One of the variants which has the same sequence to the original HpSVd-grapevine [47] we used in the following inoculation was deposited most frequently, 13 times. The major changes in the selected 45 HpSVd-grapevine variants collected from different geographical areas were at positions 26, 32, 44/45, 46, 47, 256 and 257, which were similar to those identified in the Japanese samples. There were nine additional positions that were found to vary less frequently: 105, 204, 205, 206, 207, 226, 228, 230 and 240/241 (Supp. Table S2).

### Pathogenicity of Natural HpSVd-Hop, -Grapevine, -Plum, and -Citrus Isolates in Commercial Hops over 10 Years of Persistent Infection

**Figure 2:**
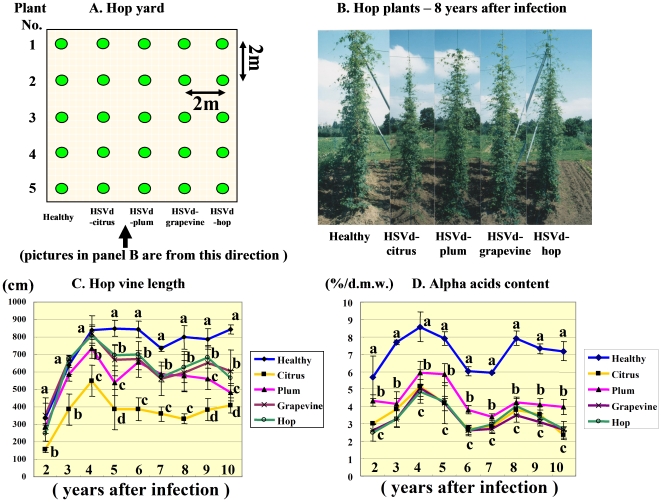
Arrangement of hop experimental plot and comparisons of the growth of infected plants. **A:** The arrangement of 25 total hop plants grown in the experimental plot. Five replicates of four natural HpSVd isolates infected into hops, in addition to 5 healthy control hop plants, were planted in 2-meter intervals. Two vines for each of the 25 hops, giving a total of 50 vines, were maintained each year. **B:** Growth of hop plants exhibited 8 years after inoculation. Photos were taken from the direction indicated in panel A. Hops infected with HpSVd-citrus showed the most severe stunting and thinning of vine volume compared to hops infected with HpSVd-plum, -grapevine, or -hop. **C**: The mean length of 10 vines measured during the 2^nd^ through 10^th^ growing seasons. Hops infected with HpSVd-grapevine and HSpVd-hop did not exhibit stunting of their vines until the 4^th^ season, and statistically significant stunting was only first observed during the 5^th^ season. In contrast, hops infected with HpSVd-citrus showed a rapid development of vine stunting. Mean vine length was only 54% that of healthy controls even in the 2^nd^ season, and further decreased to less than half by the 5^th^ season. Stunting caused by HpSVd-citrus was much more severe than that caused by either HpSVd-hop or -grapevine, while stunting caused by HpSVd-plum was intermediate between that associated with HpSVd-grapevine (or -hop) and HpSVd-citrus. **D**: Mean alpha-acid content in cones collected from the 2^nd^ through the 10^th^ growing season. Note that the alpha-acid content decreased sharply in all HpSVd-infected cones, even in the 2^nd^ season. Reduction in alpha-acid content caused by HpSVd-hop, -grapevine, and -citrus were very similar, with a 46–60% decrease during the observed years. Reduction in alpha-acid content associated with HpSVd-plum was less dramatic, being 25–45% that of the healthy controls. The lower case letters (a–d) in panels C and D are significantly different (P<0.05) according to Duncan's multiple range test.

To analyze the pathogenicity of natural HpSVd isolates during prolonged persistent infection in hops, we infected blocks of five HpSVd-free hop plants with the four major natural HpSVd isolates identified from hop (HpSVd-hop), grapevine (HpSVd-grapevine), citrus (HpSVd-citrus), and plum (HpSVd-plum). The infected hop plants were cultivated for 15 years, in addition to five healthy controls (Fig. 2A).

After 10 years, the pathogenicity of the four natural HpSVd isolates in hops was evaluated based on the presence of stunted vines and a decrease in alpha-acid content in the cones. Figure 2C shows the mean length of vines measured for the 2^nd^ through 10^th^ growing seasons. For hops infected with HpSVd-grapevine and HSpVd-hop, stunting did not appear until the 4^th^ season, and statistically significant stunting occurred during the 5^th^ season. Between the 5^th^ and 6^th^ season, mean vine lengths were 79–83% of the healthy controls, representing a mild stunted phenotype that was barely recognizable. Typical ‘hop stunt’ symptoms, including leaf curl, bent leaf, and vine stunting, were first recognized between the 7^th^ and 8^th^ seasons (Fig. 2B). In contrast, stunting of the vines infected with HpSVd-citrus progressed rapidly, with stunting, leaf bending, and severe leaf curling being apparent after only one year in the field. Mean vine length was 54% that of healthy controls in the 2^nd^ season, and further decreased by the 5^th^ season. Stunting caused by HpSVd-citrus was much more severe than that caused by either HpSVd-hop or -grape. Lateral branches were observed to droop dramatically with the edges of the leaves curled downward, while the vines became thin and their upper portions were small and sharp. This phenotype resembled the appearance of the so-called “cedar (or conifer)-shaped” hops, a typical symptom of hop stunt disease. Stunting caused by HpSVd-plum was intermediate between that associated with HpSVd-grapevine (or -hop) and HpSVd-citrus, and between the 4^th^ and 9^th^ seasons mean vine length was 65–83% that of healthy controls, statistically different from the other groups between the 3^rd^ and the 5^th^, and the 9^th^ and the 10^th^ seasons (Fig. 2C).

Alpha-acid content fluctuated year by year in each of the infected groups. In the 2^nd^, 6^th^, and 7^th^ seasons, it was relatively low (i.e., 5.7–6.0% of dry weight), even in the healthy controls. In other years, the alpha-acid content of healthy cones ranged from 7.5 to 8.5%. Data presented in Figure 2D shows that, in contrast with the slowly progressing stunted phenotype, alpha-acid content decreased sharply in all HpSVd-infected cones by the 2^nd^ season. Reductions in alpha-acid content caused by infection with HpSVd-hop, -grapevine, and -citrus were very similar, being 46–55% of the alpha-acid content of healthy controls in the 2^nd^ season, and 50–60% between the 3^rd^ and 10^th^ seasons. Reductions in alpha-acid content associated with HpSVd-plum-infected plants was less dramatic, being 25% of healthy control levels in the 2^nd^ season, and 28–45% between the 3^rd^ and the 10^th^ seasons.

### Analysis of Mutants Arising in Natural HpSVd-Hop, -Grapevine, -Plum, and -Citrus Isolates Cultivated over a 15 Year Period

#### Analysis of the original sequence in the inoculum

By using hop plants infected with the four major HpSVd isolates from hop, grapevine, plum, and citrus, the initial and final nucleotide sequences could be compared to determine the extent of molecular evolution that natural HpSVd isolates could experience over 15 years of persistent infection in hops.

**Figure 3:**
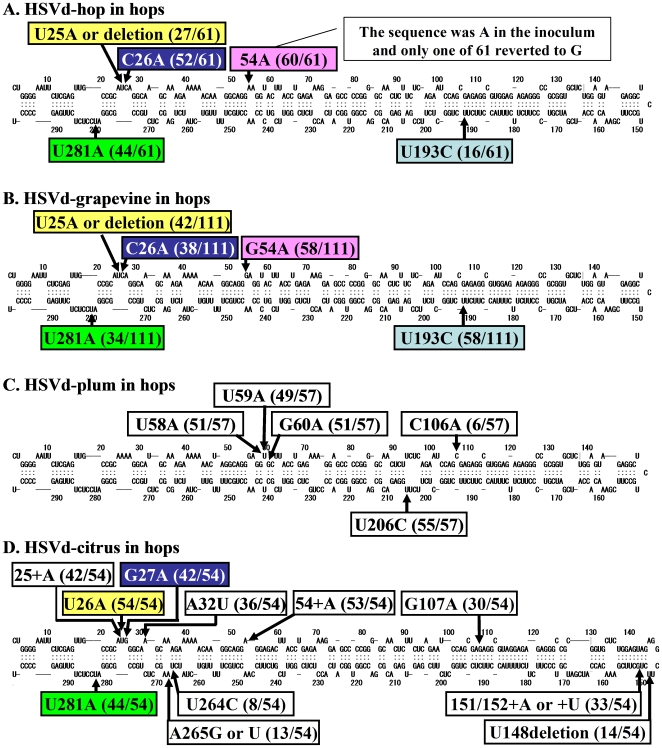
Major sequence variations emerged from four natural HpSVd isolates during 15 years of persistent infection in hops. Natural isolates were: HpSVd-hop (A), HpSVd-grapevine (B), HpSVd-plum (C), and HpSVd-citrus (D). Prediction of the secondary structure model, and location and the frequency of the mutations were drawn as in the Figure 1.

Prior to inoculation, five cDNA clones from inoculum of each of the four natural HpSVd isolates to be used were sequenced. All the sequences were identical to each other and to the sequences directly identified in the original host samples; i.e., hop, grapevine, plum and citrus. HpSVd-hop consisted of 297 nts and was identical to one of the eight major natural HpSVd variants, hJType (Table 1). HpSVd-grapevine was also 297 nts in length and was identical to the most abundant HpSVd-grapevine variant of the first group described above (Accession no. AB219944). HpSVd-plum consisted of 297 nts and was identical to the variant from plum dapple fruit (Accession no. D13764), which was also widely detected in plum (Y09350) and almond (EU937524). HpSVd-citrus consisted of 300 nts and only differed from the sequence isolated from cucumber (Accession no. X07405) by the deletion of G^53^. Predicted secondary structures of these sequences are presented in Figure 3A–D.

#### Mutation hotspots that emerged over 15 years of persistent infection

Mutations present were identified in progenies extracted independently from individual hop plants at 5, 10, 11, 14, and 15 years after the initial infection. A total of 310 randomly selected cDNA clones were sequenced. Sequences were compared to the original sequences used for the inoculum.

From the original HpSVd-hop isolate maintained in five hop plants, 22 mutation positions were identified in 67 cDNA clones (5 from the inoculum and 62 from the progeny). Although high-fidelity thermostable DNA polymerase was used, errors produced by PCR amplification could not be ruled out. Thus, single mutations were disregarded and only nucleotide positions at which at least three cDNA clones showed the same mutation were regarded as mutation hotspots. Overall, 4 mutation hotspots were identified at positions 25, 26, 193, and 281 (Fig. 3A) that were consistent between the individual hops examined. Similarly, from HpSVd-grapevine maintained in five hops, 116 cDNA clones (5 from the inoculum and 111 from the progeny) were sequenced and 25 mutation positions were identified. Using the criteria described above, five mutation hotspots were identified at positions 25, 26, 54, 193, and 281 (Fig. 3B). Four of the five mutation hotspots in HpSVd-grapevine matched those found in HpSVd-hop, as well as those in the natural HpSVd-hop population from commercial hops. The only difference between the HpSVd-hop and -grapevine isolates used for the inoculum was at nucleotide 54, which in HpSVd-hop was adenine (A) and in HpSVd-grapevine guanine (G) [47]. However, after 5 years, G^54^ was replaced by A in hops inoculated with HpSVd-grape, indicating a selection for A^54^ in hops, despite G^54^ being stably maintained in grapevines.

From the original HpSVd-plum maintained in five hop plants, 13 mutated positions were identified in 57 progeny variants analyzed. Five mutation hotspots were consistently located at positions 58, 59, 60, 106 and 206 in the individual hops examined, yet they were different from the mutations associated with HpSVd-hop and -grapevine (Fig. 3C). Positions 25, 26, 54, 193 and 281 were not altered in HpSVd-plum propagated in hops, unlike HpSVd-hop and -grapevine, indicating that HpSVd variants can undergo different adaptations in the same host plant. However, all mutations identified in HpSVd-plum are consistent with the sequences widely observed in the natural populations found in stone fruits; i.e., those from apricot in China (DQ362901), plum in Japan (AB098500), and plum in Europe (Y09352). These results suggest that the selection pressure in hops, if any, did not greatly differ from the selection pressure for HpSVd-plum in stone fruits.

From the original HpSVd-citrus propagated in five hop plants, 14 mutated positions were identified in 54 progeny variants analyzed. Eleven mutation hotspots were identified at positions 25, 26, 27, 32, 54, 107, 148/149, 150/151, 264, 265 and 281 (Fig. 3D). Only a subset of mutations at positions 25, 26, 32, 193, 265 and 281 were consistently associated with predominant mutations in natural HpSVd-hop isolates, while the remaining mutation sites were unique to HpSVd-citrus. However, most of the mutations identified have been widely observed in the natural populations in cucumber and citrus; i.e., those from cucumber pale fruit viroid in the Netherlands (X00524), citrus in Taiwan (U02527), and citrus in Europe (AF213503). Therefore, these results suggest that the host selection pressure in hops, if any, did not differ significantly from that in citrus for HpSVd-citrus.

#### Convergent evolution found in the natural HpSVd variants in hops

**Figure 4:**
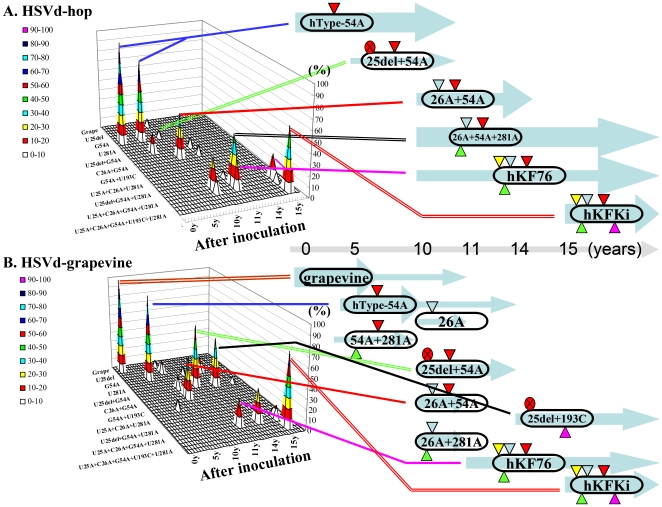
Schematic representation of the changes in major sequence variants of natural HpSVd-hop and natural HpSVd-grapevine during persistent infection in hops. The upper panel (A) indicates HpSVd-hop and the lower panel (B) indicates HpSVd-grapevine. The left figure of each panel provides a schematic representation of the changes and frequency of the resulting variants detected during 15 years of persistent infection of each of the HpSVd types in hops. The x-axis represents the adapted mutants, with only selected mutant names provided. The y-axis indicates the frequency (%) of each mutant in the population. The z-axis indicates the years after inoculation; 0 to 15 years from left to right. The right figure of each panel illustrates the changes of the predominant mutants detected. The ellipse represents the viroid molecule, with the symbols indicating: U25del (red circle with an x), U26A (yellow triangle), C26A (blue triangle), G54A (red triangle), U193C (purple triangle), and U281A (green triangle). The light blue arrows in the background indicate the time period during which the mutants were detected. A light grey arrow provides the relative time scale at the bottom of each panel. Colored lines are used to indicate correspondence with each of the main peaks in the left panel.

Changes in the predominant adaptive mutants identified were further evaluated at 5, 10, 11, 14, and 15 year intervals following the initial infection. In HpSVd-hop, all 5 cDNA clones used for the inoculum showed the same sequence as the HpSVd-hop, hJType (Table 1). Five years later, 4 of the 5 (80%) progenies analyzed had the same sequence as the original inoculum, and only 1 progeny had a deletion at nucleotide 25, designated U^25^del. Ten years later, only 1 of 14 progenies was identical to the inoculum. Six (42.9%) had a substitution at C26 (C^26^A), 4 had substitutions at both C26 and U281 (C^26^A+U^281^A), 2 had substitutions at C26, U281, and U25 (U^25^A+C^26^A+U^281^A), and 1 had a substitution at A54 (A^54^G). The latter mutation reverted the HpSVd-hop isolate to the natural HpSVd-grapevine sequence. Fifteen years later, the progenies consisted of 13 (59.1%) with 4 mutations (U^25^A+C^26^A+U^193^C+U^281^A), 5 with 2 mutations (C^26^A+U^281^A), and duplicates of 2 types of progenies with 3 mutations, (U^25^A+C^26^A+U^281^A) and (C^26^A+U^193^C+U^281^A). These mutations identified are represented schematically in Figure 4A, and in more detail in Supplementary Data Figure S2. Mutants that emerged in the sequence of HpSVd-hop transitioned from U^25^del to C^26^A, to C^26^A+U^281^A or U^25^A+C^26^A+U^281^A (which is hKF76 in natural hops), and finally resulted in (U^25^A+C^26^A+U^193^C+U^281^A). The latter represents the sequence of hKFKi, a variant found in natural hops, indicating that the four hotspot mutations at positions 25, 26, 193, and 281, accumulated slowly over the years in the progeny population. It should be noted that two predominant mutants, (U^25^A+C^26^A+U^281^A) and (U^25^A+C^26^A+U^193^C+U^281^A), emerged 10–15 years after infection and coincided with the natural HpSVd-hop isolates, hKF76 and hKFKi, respectively, both of which are predominant in commercial hops in Japan [45].

In HpSVd-grapevine, all 5 cDNA clones sequenced from the inoculum had the same sequence (HpSVd-g or hKY04-1 in Table 1). Five years later, 13 (50%) of 26 progenies sequenced contained a G^54^A mutation, making the sequences identical to HpSVd-hop, hJType. Ten years later, 7 (50%) of 14 progenies contained two mutations, U^25^del+U^281^A, while 2 contained U^25^del+G^54^A mutations, 2 contained C^26^A+G^54^A mutations, and 2 contained a C^26^A mutation. Only one conserved the original inoculum sequence. Fifteen years later, 10 (42%) of 24 progenies contained five mutations (U^25^A+C^26^A+G^54^A+U^193^C+U^281^A), 8 (33%) contained three mutations (C^26^A+G^54^A+U^281^A), and 2 contained four mutations (U^25^A+C^26^A+G^54^A+U^281^A). The substituted positions were consistent with those identified in HpSVd-hop, with mutation hotspots including positions 25, 26, 54, 193 and 281. Of note, two predominant mutants, (U^25^A+C^26^A+G^54^A+U^193^C+U^281^A) and (U^25^A+C^26^A+G^54^A+U^281^A) that emerged in year 15 coincided with natural HpSVd-hop isolates hKFKi and hKF76, respectively. These changes in HpSVd-grapevine are represented schematically in Figure 4B, and in more detail in Supplementary Data Figure S2.

For HpSVd-plum, 5 years after infection 4 of 5 cDNA clones analyzed contained the mutation U^206^C. After 10 years, 8 of 10 progenies contained four mutations (U^58^A+del^59^A+G^60^A+U^206^C), while 2 others contained another set of four mutations (U^58^A+del^59^G+G^60^A+U^206^C). Fifteen years later, all 23 progenies sequenced contained three mutations (U^58^A+del^59^A+U^206^C). Most of the mutations detected were consistent with those that are predominant among natural HpSVd-plum isolates from commercial orchards. Therefore, it was concluded that the mutations identified in HpSVd-plum were independent of host selection pressure during their incubation in hops, or alternatively, that the selection pressure on HpSVd-plum replication were similar in hop and plum plants.

In HpSVd-citrus, 4 of the 5 progenies analyzed 5 years after infection contained five mutations (U^25^C+del^26^A+G^27^A+del^54^G+del^151^U), while 1 contained six mutations (U^25^C+del^26^A+G^27^A+del^54^G+U^148^del+del^151^U). Ten years later, 7 of the 10 contained eight mutations (U^25^A+del^26^A+G^27^A+A^32^U+del^54^G+A^107^G+del^151^A+U^281^C). Fifteen years later, 5 of 11 contained six mutations (U^25^A+del^26^A+G^27^A+A^32^U+del^54^G+U^281^C), and 3 contained seven mutations (U^25^A+del^26^A+G^27^A+A^32^U+del^54^G+A^107^G+U^281^C). All of the mutations identified were unique to HpSVd-citrus.

### *De Novo* Emergence of Natural HpSVd-Hop Variants from an *In Vitro* Transcript of a Cloned HpSVd-Grapevine cDNA

To examine whether the mutants detected were created *de novo* by adaptation, or were enriched from pre-existing mutants present in the natural HpSVd-grapevine population, four HpSVd-free hop plants were infected with a transcript from an infectious cDNA clone of HpSVd-grapevine. These four plants were then cultivated for 10 years. The infectious HpSVd-grapevine cDNA clone used for these experiments is the same sequence previously referred to as HpSVd-grapevine (AB219944, Table 1).

A total of 193 cDNA clones were sequenced, which included 46–52 clones from each of the four original plants infected with HpSVd-grapevine RNA. Sixty-six mutation positions were identified, including five mutation hotspots: U^25^A, C^26^A, G^54^A, U^193^C and U^281^A. All the mutations identified were present in the natural HpSVd-grapevine propagated in hops (Fig. 3B). The frequency of an additional insertion mutation between positions 44 and 45 was detected in only 8 of 193 clones.

**Figure 5:**
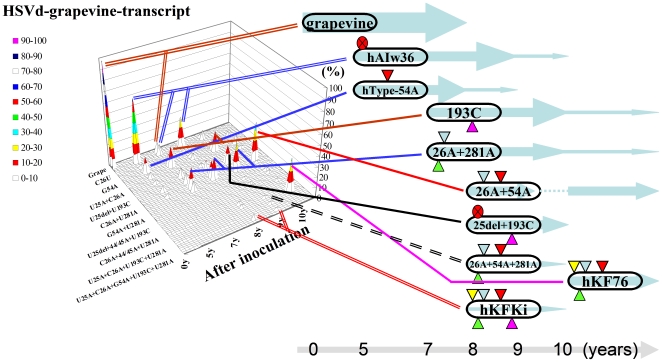
Schematic representation of the changes in major sequence variants of the HpSVd-grapevine transcript during persistent infection in hops. The left and the right parts were drawn as in the Figure 4. The z-axis indicates the years after inoculation; 0 to 10 years from left to right.

All the mutants detected over 10 years of persistent infection in hops, and their relative ratios in the population, are schematically presented in Figure 5, and in more detail in Supplementary Data Figure S2. Mutants that emerged from the HpSVd-grapevine transcript included: U^25^del (63%) and G^54^A (13%) after 5 years, and C^26^A+G^54^A (38%) and U^25^A+C^26^A+G^54^A+U^281^A (33%) after 10 years. Mutants (C^26^A+G^54^A) and (U^25^A+C^26^A+G^54^A+U^281^A) were also identified as major adaptive mutants in the natural HpSVd-grapevine and HpSVd-hop isolates during persistent infection in hops. Furthermore, mutants (G^54^A), (U^25^del), (C^26^A+U^281^A), (U^25^A+C^26^A+G^54^A+U^281^A), and (U^25^A+C^26^A+G^54^A+U^193^C+U^291^A) are identical to the natural HpSVd variants endemic in commercial hops: hJType, hAIw36, hAIw5, hKF76, and hKFKi, respectively [45]. In combination, these data indicate that the adaptive mutants identified in this study developed *de novo* in hops by host selection/pathogen adaptation.

### Mutation Frequency of HpSVd Isolates in Hops over 15 Years of Persistent Infection

The mutation frequency (nucleotides/site/year) of four natural HpSVd isolates grown in duplicates in hop plants were calculated. All mutations, including single mutations, were included in this calculation. The mutation frequency of HpSVd-hop was 1.3×10^−4^, 5.5×10^−4^, and 6.7–9.6×10^−4^ at 5, 10, and 15 year intervals, respectively. In HpSVd-grapevine, the mutation frequency was 8.1×10^−4^–1.0×10^−3^, 8.1×10^−4^, and 7.2×10^−4^–1.1×10^−3^ at 5, 10, and 15 year intervals, respectively. The mutation frequency of HpSVd-grapevine was higher than that of HpSVd-hop, especially after the first 5 years, which can be attributed to the G-to-A substitution that occurred at nucleotide 54. However, the mutation frequency was even higher in HpSVd-citrus, with values of 3.4×10^−3^, 2.5×10^−3^, and 2.2–1.4×10^−3^ at 5, 10, and 15 year intervals, respectively. The mutation frequency of HpSVd-plum was between that of HpSVd-hop and -citrus. In summary, the mutation frequency of the 4 HpSVd isolates incubated in hops for 15 years ranged from 1.3×10^−4^ to 3.4×10^−3^. An extremely high mutation rate (ca. 2.5×10^−3^) has been identified for a viroid belonging to the family *Avsunviroidae*, the members of which replicate in chloroplasts [50]. It is not possible to compare these two data, because the mutation frequency in the present work means changes incorporated in a population during a point in time (nucleotide/site/year), on the other hand, the value identified in *Avsunviroidae* means a mutation rate per site and replication cycle. Nevertheless, the value identified in the present work was comparable to the estimated per nucleotide mutation rate presented in the literature [19] for *Citrus bent leaf viroid* (ca. 2−3×10^−3^), the family *Pospiviroidae*, and was 1/2–1/3 times lower than that of *Avsunviroidae* (ca. 5–7×10^−3^). Furthermore, the mutation frequency of HpSVd in the present work was comparable to that reported for plant RNA viruses, such as *Cucumber mosaic virus* (0.5–2.5×10^−3^ nucleotides/site/year, [51], [52]) and *Tobacco mosaic virus* (0.5–2.0×10^−3^, [51], [52]), or for *Begomovirus* of the family *Geminiviridae*, such as Tomato yellow leaf curl China virus (2.6–5.3×10^−4^, [51], [53]) and *Maize streak virus* (2–3×10^−4^, [51], [54]), which have circular single-stranded DNA genomes replicating inside the nucleus. Namely, the data in our experiments suggested that members of the family *Pospiviroidae* can also mutate quickly when introduced into new host species.

## Discussion

### Convergent Evolution of Hop Stunt Viroid Isolates during Prolonged Persistent Infection in Hops

**Figure 6:**
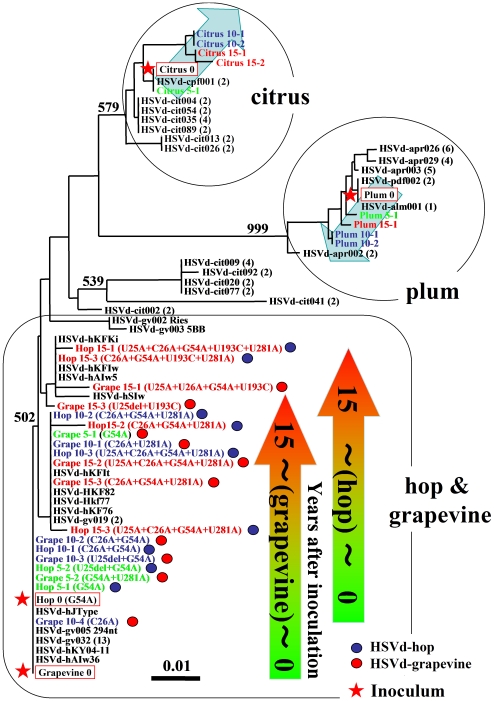
Phylogenetic relationship of the predominant mutants that emerged during the persistent infection of the four natural HpSVd isolates in hops compared to major sequence variants deposited in the SubViral RNA database. The predominant mutants presented in Figures 4 and 5 were aligned with the major HpSVd variants deposited in the Subviral RNA database in order to generate a Neighbor-Joining tree. Original variants present in the inoculum are outlined in red boxes and labeled with a red star. Variants shown in green, blue, and red lettering are those that were predominant at the 5, 10, and 15 year intervals after inoculation, respectively. Variants accompanied by a blue or red circle indicate that they are derived from HpSVd-hop or from HpSVd-grapevine, respectively. Black lettering indicates the major natural HpSVd variants currently present in hop fields. Colored (green to red) arrows in the figure represent the direction of changes detected in HpSVd isolates from hop and grapevine, which converged to the acquisition of 5 mutations at positions 25, 26, 54, 193, and 281. These mutations are the same for HpSVd isolates that are endemic in hop production fields in Japan. The predominant mutants that emerged from HpSVd-plum and HpSVd-citrus during infection in hops were similar to the natural variants currently found in plum and citrus, respectively. The grey arrows in the citrus and the plum represent the direction of changes detected in hops during infection. Numbers provided in parentheses indicate the number of identical sequences deposited in the SubViral RNA database, and the bar (0.01) represents the distance of 1 change in 100 nucleotides. The numbers in the node indicate the bootstrap value (i.e. 1000 replicates).

Both a natural HpSVd-hop and -grapevine variant, in addition to an *in vitro* transcript of HpSVd-grapevine, were introduced into hops and cultivated over 15 years. The resulting progenies from HpSVd-hop and -grapevine presented changes at nucleotide positions 25, 26, 193 and 281, and 25, 26, 54, 193 and 281, respectively. Similarly, the progeny of a natural plum variant contained mutations at positions 58, 59, 60 and 206, while that of a natural citrus variant contained mutations at positions 25, 26, 27, 32, 54, 107, 148/149, 150/151, 264, 265 and 281. Mutation hotspots were reproducibly detected in multiple hop plants analyzed, suggesting that positive host-selection pressure existed. Figure 6 represents the phylogenetic relationship of the predominant mutant variants that emerged from the four types of natural HpSVd isolates maintained as persistent infections in hops for 5, 10, and 15 year intervals compared to the major HpSVd variants deposited in the Subviral RNA database. HpSVd isolates from hop and grapevine exhibited similar changes (as indicated by arrows in Fig. 6) at positions 25, 26, 54, 193 and 281. Two distantly-related variants of plum and citrus, with ∼93% and ∼92% sequence identity with the grapevine variant, respectively, presented mutations in different positions from those of hop and grapevine. These results indicate that the host-selection pressure was not consistent between hosts, and correspondingly variants adapted differently. However, since the predominant mutants obtained from persistent infection in hops with HpSVd-plum and HpSVd-citrus were similar to the natural variants found in plum and citrus, respectively, the host selection pressure on HpSVd appears to be similar between hop and plum, and hop and citrus.

**Figure 7:**
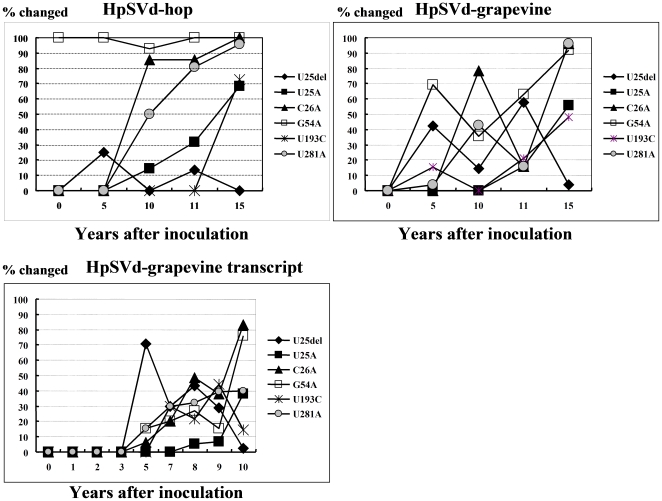
Accumulation of HpSVd mutations in five hotspots during persistent infection in hops. Adaptive mutants from each of the 5 mutation hotspots emerged similarly in the HpSVd-hop and HpSVd-grapevine sequences, as well as in the cloned HpSVd-grapevine transcript. The U^25^del mutation appeared soon after infection, was quickly replaced by U^25^A, and then disappeared from the population. The mutations U^26^A, G^54^A, and U^281^A accumulated gradually and eventually were present in >99% of the variants sequenced. The U^25^A and U^193^C mutations emerged in the later years of the infection, and reached ∼50–70% of the progeny population after 15 years.

During the adaptation of the natural or *in vitro* transcript of HpSVd-grapevine in hops, mutations only occurred in five hotspots at positions 25, 26, 54, 193 and 281. In general, the U^25^del was found to appear first in the population and then it was gradually replaced by U^25^A (Fig. 7). The mutations U^26^A, G^54^A and U^281^A were detected in almost all progeny, whereas the U^25^A and U^193^C mutations appeared relatively slowly and were maintained by year 15 by at most 50–70% of the population (Fig. 7). We hypothesize that these mutations gradually accumulated via multiple pathways, with variants containing all five hotspot mutations (U^25^A+C^26^A+G^54^A+U^193^C+U^281^A), or 4 of the 5 mutations (U^25^A+C^26^A+G^54^A+U^281^A), eventually becoming predominant in the population. These two variants were also identical to the HpSVd variants identified in the natural HpSVd-hop population, hKFKi and hKF76, respectively [45].

The pathogenicity associated with these mutations was also enhanced. The typical ‘hop stunt’ symptoms were visible 7 years after infection with HpSVd-grapevine, when variants with multiple hotspot mutations predominated in the population. In the mean time, positive correlation can not be found between accumulation of the mutations and the thermal stabilities of the variants by Mfold analysis [55, data not shown]. Namely, 4 of the 5 mutation hotspots (i.e. 25, 26, 54 and 281) involved A substitutions and were located in loops of the predicted secondary structure. As such, none of them appear to cause structural changes. The mutation at position 193 that appeared years later involved a C substitution located in a stem. However, by changing a G:U pair into a G:C pair, the mutation is predicted to stabilize this stem.

By setting up a parallel experiment using infectious *in vitro* transcripts of a cloned HpSVd-grapevine variant, it was confirmed the emergence of mutations identical to those found in natural HpSVd-grapevine and -hop variants. Therefore, the natural HpSVd-hop variants found in commercial hops are *de novo* mutants generated by the same process through which HpSVd-grapevine adapted to a hop host, and not mutants selected from minor variants pre-existing in the original inoculum. Similar events are also reported in several plant viruses and viroids [19], [20], [51]–[53]; i.e., transfer of virus or viroid from one host to another results in the appearance of new sequence variants.

### A Widespread HpSVd Variant in Grapevines Causes Hop Stunt Epidemics

Among the natural HpSVd isolates, the nucleotide sequence of hop and grapevine isolates was found to be similar, which may be linked with the overlapping cultivation areas for these two crops in Japan [34], [36], [47]. In the analysis of the genetic diversity of HpSVd variants endemic in commercial hops in Northern Japan, it was observed that all the sequenced hop isolates formed a phylogenetic cluster with those from grapevines [45]. These findings suggested that the HpSVd present in hops may have originated from grapevines. Based on the convergent evolution observed in the adaptation of HpSVd isolates to hops in these experiments, the possible origins of hop stunt epidemics can now be discussed.

Our studies demonstrate that 15 years of persistent infection in hops resulted in the evolution of natural HpSVd-grapevine variants into HpSVd-hop variants identical to those currently responsible for the epidemic in commercial hops in Japan. Parallel experiments using HpSVd-citrus and -plum revealed that they also evolve in hops, but these variations detected were not consistent with those found in the natural HpSVd population in commercial hops. Rather, they were more similar to those found in the natural HpSVd populations in commercial citrus and stone fruits. Consequently, our data indicate that HpSVd-grapevine is the origin of the hop stunt epidemics. In addition, the major sequence variants hKY04-1 and 04-7, detected in commercial hop gardens in 2004, were found to be identical to HpSVd-grapevine and its U25-deletion mutant hAIw36, respectively. Given that both HpSVd-grapevine and the U^25^del mutant were unstable in hop plants in our experiments and soon disappeared from the population, these data suggest that HpSVd transmission from grapevines to hops is ongoing in hop-growing areas of Japan.

Similarly, Xinjiang-derived HpSVd-hop isolates contain the same 5 hotspot mutations identified in HpSVd-grapevine variants that adapted to hops, with a major sequence variant identified being identical to the Japanese variant hKFKi. Regarding the recent outbreak in the state of Washington in the US, the major variant was also identical to the hKFKi variant, suggesting that HpSVd variants in the US and Chinese epidemics derived from HpSVd-grapevine, and the hKFKi variant is a partially adapted mutant. Furthermore, the hop stunt epidemics in China and the US may have also originated from inter-specific transmission of HpSVd from cultivated grapevines to hops, since both regions also produce grapes. This circumstantial evidence, together with the observation that commercial grapevines commonly harbor latent HpSVd, supports the potential for the hop stunt epidemics to occur in other areas of the world.

Viroids sometimes infect crops asymptomatically, as in the case of the hop stunt viroid in grapevines and citrus. A variety of crops, including fruit trees, are vegetatively propagated for widespread distribution overseas. With the globalization of agriculture, it is easier for viroids to become widely distributed and introduced into new environments and induce disease epidemics. This is the case for several *Pospiviroids*, such as potato spindle tuber and tomato chlorotic dwarf viroid, which have been recently reported in ornamentals in the family of *Solanaceae* [56]–[58]. Potato spindle tuber was the first viroid discovered and is an extremely dangerous pathogen in potato [1], [3]. However, this agent replicates asymptomatically in other hosts of the same family, and thus is able to spread without obvious symptoms. A similar situation now faces cultivated hops and grapevines. Therefore, it is essential that sensitive and simpler detection techniques be developed for the diagnosis of viroids at production sites in order to facilitate the protection of uninfected crops.

## Materials and Methods

### Natural HpSVd Isolates in Hops and Grapevines

Natural HpSVd isolates were collected from commercial hops (*Humulus lupulus*) in Japan between 1992 and 2004, and from commercial hops in Xinjiang, China in 2007. HpSVd isolates were also collected from cultivated grapevines (*Vitis vinifera*) grown in three nursery yards and several commercial vineyards in Japan between 1992 and 2003.

### Infection of Hops with Four Natural HpSVd Isolates and an *In Vitro* Transcript of HpSVd-Grapevine

Low molecular weight (LMW) RNAs were extracted from hop [29], grapevine [47], citrus [37], and plum [38] plants to infect commercial hop plants with four types of natural HpSVd isolates. Since these four crops commonly harbor multiple viroid species, including HpSVd, the LMW-RNAs were first inoculated into cucumber (*Cucumis sativus*, cv. Suyo), which is an indicator and selective host for HpSVd, in order to eliminate the other viroid species that may have been present in the collected inoculum. The cucumber plants were incubated for four weeks until they exhibited an HpSVd-specific phenotype, which includes leaf curling and stunting, before LMW-RNAs were extracted. Further detection of viroid species present using sequential polyacrylamide gel electrophoresis (PAGE) [47], Northern hybridization [46], and reverse transcription – polymerase chain reaction (RT-PCR) only identified HpSVd. Full length cDNAs amplified by RT-PCR directly from the original host crops (i.e., hop, grapevine, plum and citrus), as well as from the infected cucumber plants, were further processed for cloning and sequencing. Five full-length cDNA clones from each of the original hosts and infected cucumbers were found to be identical with each other, indicating that the sequences were not changed before and after their passage through cucumbers. Namely, incubation of the four HSVd-isolates in cucumber for four weeks successfully isolated HSVd only without causing any detectable filtering effect upon the genetic populations in the inoculum. LMW-RNA preparations (400 µg/ml in 0.1 M Tris-HCl (pH 7.5)–0.1% bentonite) were used for the inoculation of virus-free hop cuttings (cv. Kirin II, 50 µl per plant) in the spring of 1993.

A recombinant plasmid (pBS-HpSVd-4U) containing four tandem repeat copies of the HpSVd-grapevine cDNA was inserted in the Bam HI site of pBluescript II SK(−) (Stratagene) for RNA transcription by T7 RNA polymerase (Invitrogen, Carlsbad, CA). The infectious HpSVd–grapevine transcript (10 ng/µl in 0.05 M Tris-HCl (pH 7.5)–0.1% bentonite) was used for the infection of virus-free hops (cv. Kirin II, 20 µl per plant) in the spring of 1998.

### Hop Cultivation and Harvest

Five replicates of each of the four natural HpSVd isolates inoculated into hops, in addition to five healthy controls giving a total of 25 hops, were planted in an experimental plot (10 m×10 m, Fig. 1A) in April 1994. These hops were cultivated and maintained for 15 years. Plants infected with the HpSVd–grapevine transcripts were grown in individual pots and maintained in a greenhouse (ca.10–30°C) from April to November for 10 years.

Between 1994 (2^nd^ growing season) and 2002 (10^th^ growing season), two vines per plants originally infected with replicates of the four natural HpSVd isolates were selected for cultivation, resulting in a total of 50 vines. These vines were harvested in early September from 1993 to 2002 to measure the length of vine, while cones were also harvested from each of the 25 hops for alpha-acid analysis.

### Viroid Extraction and Molecular Hybridization

Every summer (mid-August), 1–5 g of fresh mature leaves were collected from each of the 25 hop plants for extraction of LMW-RNAs as previously described [46]. The LMW-RNA samples were subjected to molecular hybridization, RT-PCR, cDNA cloning, and sequencing analysis.

### RT–PCR, cDNA Cloning, Sequencing, and Phylogenetic Analysis

Reverse transcription of LMW-RNAs was performed using primer HSV-105M (5′-GCTGGATTCTGAGAAGAGTT-3′). Full-length HpSVd cDNAs were amplified by PCR using two primers: HSV-78P (5′-AACCCGGGGCAACTCTTCTC-3′) and HSV-83M (5′-AACCCGGGGCTCCTTTCTCA-3′) [45]. Full-length PCR products (∼300 bp) were cloned into the pGEM-T vector system (Promega, Tokyo, Japan) and sequenced. The sequence covered by the PCR primers (nts 78–105) were determined using direct sequencing of the RT-PCR products obtained with primers HSV-7P (5′-AATTCTCGAGTTGCCGC-3′) and HSV-220M (5′-CGAACCGAGAGGTGATGCCA-3′). Nucleotide sequencing was performed using Li-cor 4000I (Li-cor) or ABI prism 310 (ABI) DNA sequencers, or determined by Macrogen (Seoul, Korea). The HpSVd sequences obtained were aligned using ClustalW (Ver.1.74, DNA Data Bank of Japan; DDBJ: http://www.ddbj.nig.ac.jp).

## Supporting Information

Figure S1 Two types of HpSVd-grapevine
(0.56 MB TIF)

Figure S2 The transitions and the frequency of all the sequence variations detected in the natural HpSVd-hop, natural HpSVd-grape, and HpSVd-grape RNA transcript during the persistent infection in hops were shown by percentage in the population. All the original sequence variants in the inocula were gradually disappeared and, in stead, various adaptive sequence variants were predominated over the years. The predominant sequence variants with shade; i.e., (U25del), (G54A), (U193C), (U25A+C26A+G54A+U281A), and (U25A+C26A+G54A+C193U+U281A) were identical to the predominant sequences of natural HpSVd-hop isolates actually epidemic in the commercial hops in Japan; i.e., hAIw36, hJType, hKF76, and hKFKi, respectively.
(0.29 MB TIF)

Table S1 Multiple sequence alignment of natural HpSVd-grapevine sequences in Japan
(0.05 MB DOC)

Table S2 Multiple sequence alignment of HpSVd-grapevine deposited in SubViral RNA database
(0.07 MB DOC)
