# Supplementary figures and images for: Cultivated Grapevines Represent a Symptomless Reservoir for the Transmission of Hop Stunt Viroid to Hop Crops: 15 Years of Evolutionary Analysis
Source: PLoS One. 2009 Dec 24;4(12):e8386. doi: 10.1371/journal.pone.0008386 (PMC2793511; doi:10.1371/journal.pone.0008386)

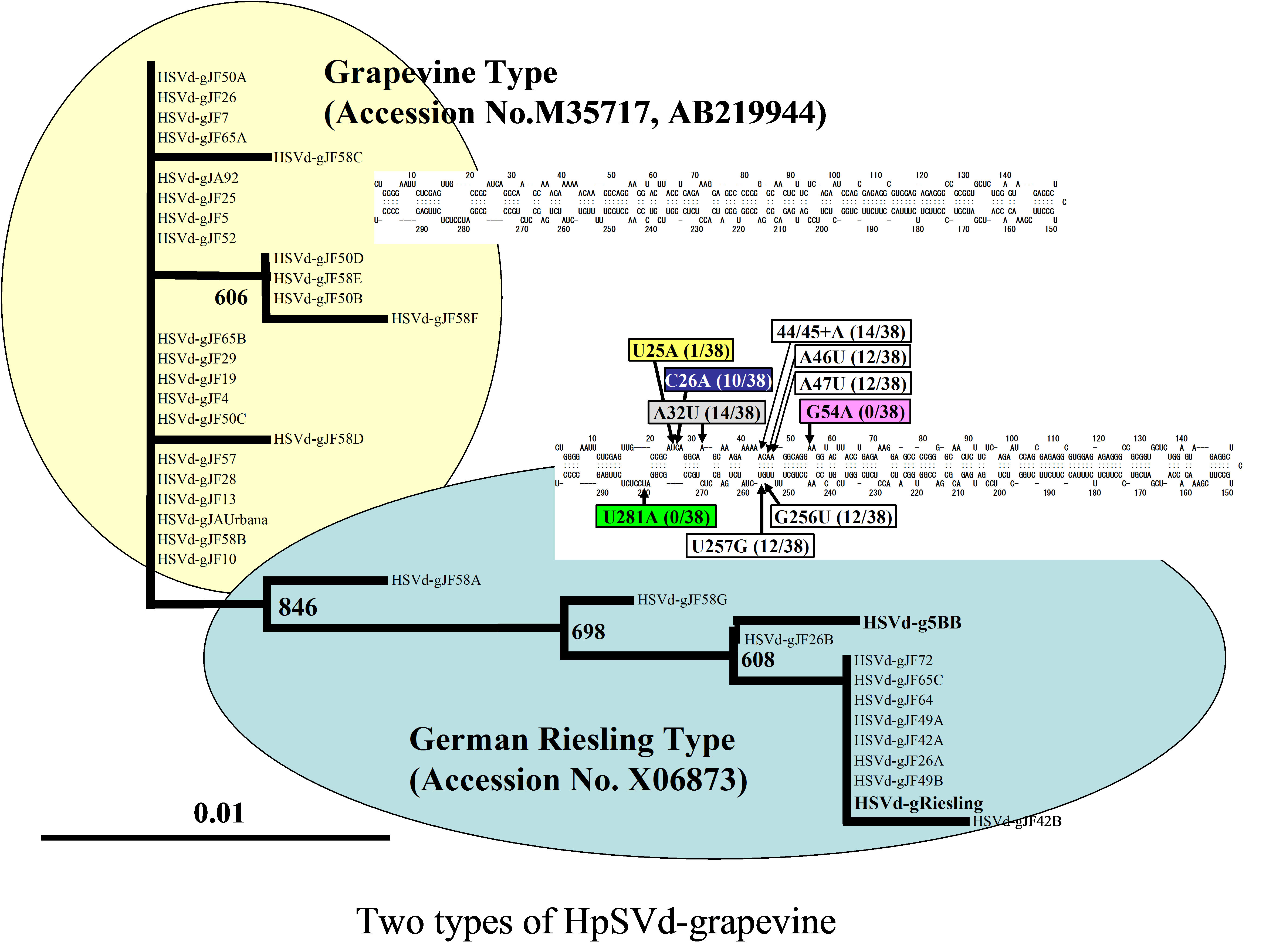

Supplement: Figure S1 — Two types of HpSVd-grapevine (0.56 MB TIF) [file pone.0008386.s001.tif]

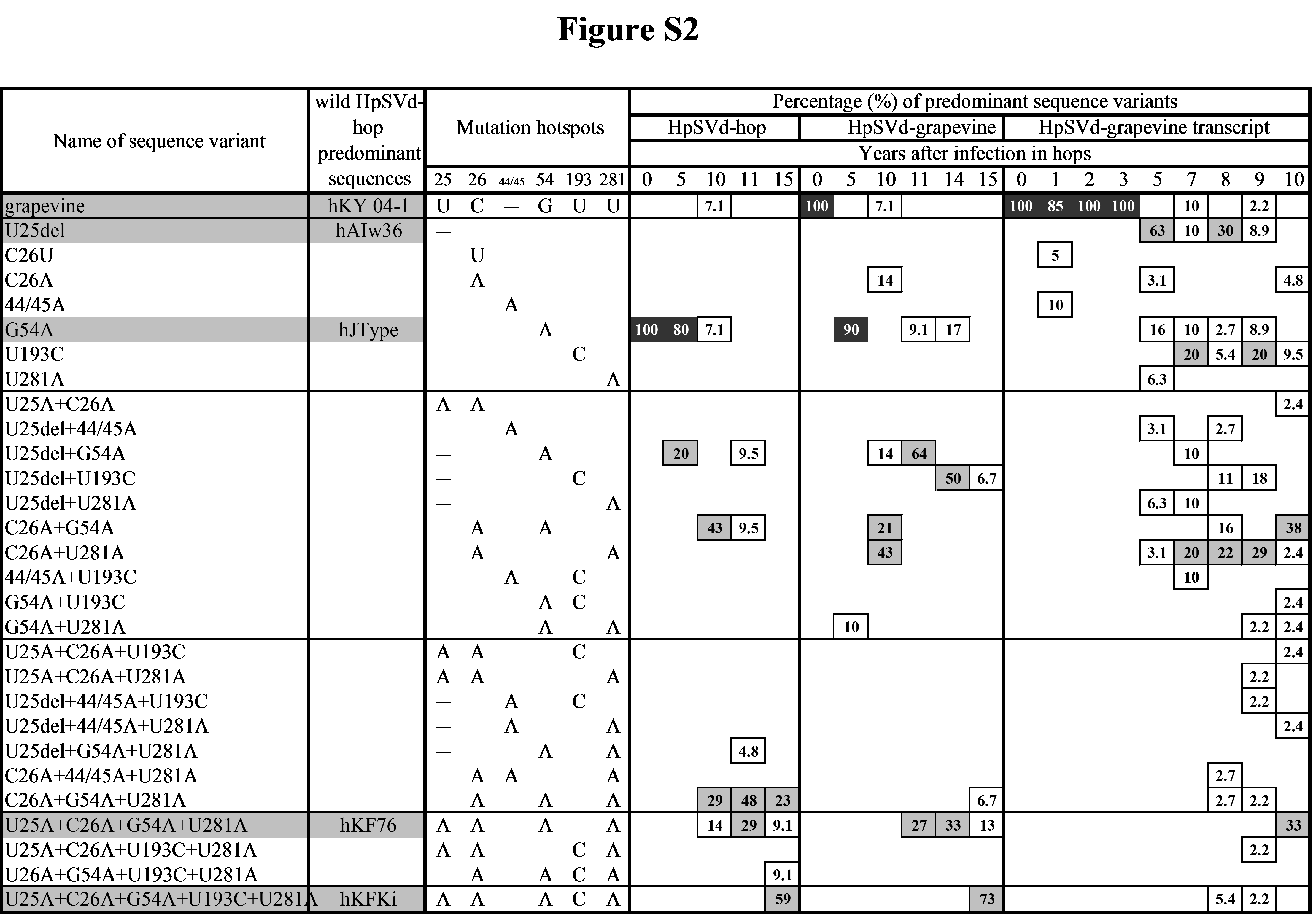

Supplement: Figure S2 — The transitions and the frequency of all the sequence variations detected in the natural HpSVd-hop, natural HpSVd-grape, and HpSVd-grape RNA transcript during the persistent infection in hops were shown by percentage in the population. All the original sequence variants in the inocula were gradually disappeared and, in stead, various adaptive sequence variants were predominated over the years. The predominant sequence variants with shade; i.e., (U25del), (G54A), (U193C), (U25A+C26A+G54A+U281A), and (U25A+C26A+G54A+C193U+U281A) were identical to the predominant sequences of natural HpSVd-hop isolates actually epidemic in the commercial hops in Japan; i.e., hAIw36, hJType, hKF76, and hKFKi, respectively. (0.29 MB TIF) [file pone.0008386.s002.tif]
