# Supplementary material for: Cultivated Grapevines Represent a Symptomless Reservoir for the Transmission of Hop Stunt Viroid to Hop Crops: 15 Years of Evolutionary Analysis
Source: PLoS One. 2009 Dec 24;4(12):e8386. doi: 10.1371/journal.pone.0008386 (PMC2793511; doi:10.1371/journal.pone.0008386)
Supplement: Table S1 — Multiple sequence alignment of natural HpSVd-grapevine sequences in Japan (0.05 MB DOC) [file pone.0008386.s003.doc]

**Multiple sequence alignment of natural HpSVd-grapevine predominant sequences in Japan**

**Name** **Sequence**

HSVd-gJF10 CTGGGGAATTCTCGAGTTGCCGCATCAGGCAAGCAAAGAAAAAA-CAAGGCAGGGAGGTA

HSVd-gJF58B CTGGGGAATTCTCGAGTTGCCGCATCAGGCAAGCAAAGAAAAAA-CAAGGCAGGGAGGTA

HSVd-gJF50C CTGGGGAATTCTCGAGTTGCCGCATCAGGCAAGCAAAGAAAAAA-CAAGGCAGGGAGGTA

HSVd-gJF52 CTGGGGAATTCTCGAGTTGCCGCATCAGGCAAGCAAAGAAAAAA-CAAGGCAGGGAGGTA

HSVd-gJF65A CTGGGGAATTCTCGAGTTGCCGCATCAGGCAAGCAAAGAAAAAA-CAAGGCAGGGAGGTA

HSVd-gJAUrbana CTGGGGAATTCTCGAGTTGCCGCATCAGGCAAGCAAAGAAAAAA-CAAGGCAGGGAGGTA

HSVd-gJF4 CTGGGGAATTCTCGAGTTGCCGCATCAGGCAAGCAAAGAAAAAA-CAAGGCAGGGAGGTA

HSVd-gJF5 CTGGGGAATTCTCGAGTTGCCGCATCAGGCAAGCAAAGAAAAAA-CAAGGCAGGGAGGTA

HSVd-gJF7 CTGGGGAATTCTCGAGTTGCCGCATCAGGCAAGCAAAGAAAAAA-CAAGGCAGGGAGGTA

HSVd-gJF13 CTGGGGAATTCTCGAGTTGCCGCATCAGGCAAGCAAAGAAAAAA-CAAGGCAGGGAGGTA

HSVd-gJF19 CTGGGGAATTCTCGAGTTGCCGCATCAGGCAAGCAAAGAAAAAA-CAAGGCAGGGAGGTA

HSVd-gJF25 CTGGGGAATTCTCGAGTTGCCGCATCAGGCAAGCAAAGAAAAAA-CAAGGCAGGGAGGTA

HSVd-gJF26 CTGGGGAATTCTCGAGTTGCCGCATCAGGCAAGCAAAGAAAAAA-CAAGGCAGGGAGGTA

HSVd-gJF28 CTGGGGAATTCTCGAGTTGCCGCATCAGGCAAGCAAAGAAAAAA-CAAGGCAGGGAGGTA

HSVd-gJF29 CTGGGGAATTCTCGAGTTGCCGCATCAGGCAAGCAAAGAAAAAA-CAAGGCAGGGAGGTA

HSVd-gJA92 CTGGGGAATTCTCGAGTTGCCGCATCAGGCAAGCAAAGAAAAAA-CAAGGCAGGGAGGTA

HSVd-gJF58D CTGGGGAATTCTCGAGTTGCCGCATCAGGCAAGCAAAGAAAAAA-CAAGGCAGGGAGGTA

HSVd-gJF58C CTGGGGAATTCTCGAGTTGCCGCATCAGGCAAGCAAAGAAAAAA-CAAGGCAGGGAGGTA

HSVd-gJF50A CTGGGGAATTCTCGAGTTGCCGCATCAGGCAAGCAAAGAAAAAAACAAGGCAGGGAGGTA

HSVd-gJF57 CTGGGGAATTCTCGAGTTGCCGCATCAGGCAAGCAAAGAAAAAAA-AAGGCAGGGAGGTA

HSVd-gJF65B CTGGGGAATTCTCGAGTTGCCGCATCAGGCAAGCAAAGAAAAAAACAAGGCAGGGAGGTA

HSVd-gJF50B CTGGGGAATTCTCGAGTTGCCGCATCAGGCAAGCAAAGAAAAAA-CAAGGCAGGGAGGTA

HSVd-gJF58F CTGGGGAATTCTCGAGTTGCCGCATCAGGCAAGCAAAGAAAAAA-CAAGGCAGGGAGGTA

HSVd-gJF58E CTGGGGAATTCTCGAGTTGCCGCATCAGGCAAGCAAAGAAAAAA-CAAGGCAGGGAGGTA

HSVd-gJF50D CTGGGGAATTCTCGAGTTGCCGCATCAGGCAAGCAAAGAAAAAAACAAGGCAGGGAGGTA

HSVd-gJF58A CTGGGGAATTCTCGAGTTGCCGCATCAGGCAAGCAAAGAAAAAA-CAAGGCAGGGAGGTA

HSVd-g5BB CTGGGGAATTCTCGAGTTGCCGCATCAGGCATGCAAAGAAAAAAACTTCGCAGGGAGGTA

HSVd-gJF26B CTGGGGAATTCTCGAGTTGCCGCATCAGGCATGCAAAGAAAAAAACTTGGCAGGGAGGTA

HSVd-gRiesling CTGGGGAATTCTCGAGTTGCCGCATAAGGCATGCAAAGAAAAAAACTTGGCAGGGAGGTA

HSVd-gJF49B CTGGGGAATTCTCGAGTTGCCGCATAAGGCATGCAAAGAAAAAA-CTTGGCAGGGAGGTA

HSVd-gJF42B CTGGGGAATTCTCGAGTTGCCGCAAAAGGCATGCAAAGAAAAAAACTTGGCAGGGAGGTA

HSVd-gJF26A CTGGGGAATTCTCGAGTTGCCGCATAAGGCATGCAAAGAAAAAAACTTGGCAGGGAGGTA

HSVd-gJF42A CTGGGGAATTCTCGAGTTGCCGCATAAGGCATGCAAAGAAAAAAACTTGGCAGGGAGGTA

HSVd-gJF49A CTGGGGAATTCTCGAGTTGCCGCATAAGGCATGCAAAGAAAAAAACTTGGCAGGGAGGTA

HSVd-gJF64 CTGGGGAATTCTCGAGTTGCCGCATAAGGCATGCAAAGAAAAAAACTTGGCAGGGAGGTA

HSVd-gJF65C CTGGGGAATTCTCGAGTTGCCGCATAAGGCATGCAAAGAAAAAAACTTGGCAGGGAGGTA

HSVd-gJF72 CTGGGGAATTCTCGAGTTGCCGCATAAGGCATGCAAAGAAAAAAACTTGGCAGGGAGGTA

HSVd-gJF58G CTGGGGAATTCTCGAGTTGCCGCATAAGGCATGCAAAGAAAAAA-CTTGGCAGGGAGGTA

************************ ***** ************ ***********

**Nucleotide position 26 32 44/45, 46-47**

HSVd-gJF10 CTTACCTGAGAAAGGAGCCCCGGGGCAACTCTTCTCAGAATCCAGCGAGAGGCGTGGAGA

HSVd-gJF58B CTTACCTGAGAAAGGAGCCCCGGGGCAACTCTTCTCAGAATCCAGCGAGAGGCGTGGAGA

HSVd-gJF50C CTTACCTGAGAAAGGAGCCCCGGGGCAACTCTTCTCAGAATCCAGCGAGAGGCGTGGAGA

HSVd-gJF52 CTTACCTGAGAAAGGAGCCCCGGGGCAACTCTTCTCAGAATCCAGCGAGAGGCGTGGAGA

HSVd-gJF65A CTTACCTGAGAAAGGAGCCCCGGGGCAACTCTTCTCAGAATCCAGCGAGAGGCGTGGAGA

HSVd-gJAUrbana CTTACCTGAGAAAGGAGCCCCGGGGCAACTCTTCTCAGAATCCAGCGAGAGGCGTGGAGA

HSVd-gJF4 CTTACCTGAGAAAGGAGCCCCGGGGCAACTCTTCTCAGAATCCAGCGAGAGGCGTGGAGA

HSVd-gJF5 CTTACCTGAGAAAGGAGCCCCGGGGCAACTCTTCTCAGAATCCAGCGAGAGGCGTGGAGA

HSVd-gJF7 CTTACCTGAGAAAGGAGCCCCGGGGCAACTCTTCTCAGAATCCAGCGAGAGGCGTGGAGA

HSVd-gJF13 CTTACCTGAGAAAGGAGCCCCGGGGCAACTCTTCTCAGAATCCAGCGAGAGGCGTGGAGA

HSVd-gJF19 CTTACCTGAGAAAGGAGCCCCGGGGCAACTCTTCTCAGAATCCAGCGAGAGGCGTGGAGA

HSVd-gJF25 CTTACCTGAGAAAGGAGCCCCGGGGCAACTCTTCTCAGAATCCAGCGAGAGGCGTGGAGA

HSVd-gJF26 CTTACCTGAGAAAGGAGCCCCGGGGCAACTCTTCTCAGAATCCAGCGAGAGGCGTGGAGA

HSVd-gJF28 CTTACCTGAGAAAGGAGCCCCGGGGCAACTCTTCTCAGAATCCAGCGAGAGGCGTGGAGA

HSVd-gJF29 CTTACCTGAGAAAGGAGCCCCGGGGCAACTCTTCTCAGAATCCAGCGAGAGGCGTGGAGA

HSVd-gJA92 CTTACCTGAGAAAGGAGCCCCGGGGCAACTCTTCTCAGAATCCAGCGAGAGGCGTGGAGA

HSVd-gJF58D CTTACCTGAGAAAGGAGCCCCGGGGCAACTCTTCTCAGAATCCAGCGAGAGGCGTGGAGA

HSVd-gJF58C CTTACCTGAGAAAGGAGCCCCGGGGCAACTCTTCTCAGAATCCAGCGAGAGGCATGGAGA

HSVd-gJF50A CTTACCTGAGAAAGGAGCCCCGGGGCAACTCTTCTCAGAATCCAGCGAGAGGCGTGGAGA

HSVd-gJF57 CTTACCTGAGAAAGGAGCCCCGGGGCAACTCTTCTCAGAATCCAGCGAGAGGCGTGGAGA

HSVd-gJF65B CTTACCTGAGAAAGGAGCCCCGGGGCAACTCTTCTCAGAATCCAGCGAGAGGCGTGGAGA

HSVd-gJF50B CTTACCTGAGAAAGGAGCCCCGGGGCAACTCTTCTCAGAATCCAGCGAGAGGCGTGGAGA

HSVd-gJF58F CTTACCTGAGAAAGGAGCCCCGGGGCAACTCTTCTCAGAATCCAGCGAGAGGCGTGGAGA

HSVd-gJF58E CTTACCTGAGAAAGGAGCCCCGGGGCAACTCTTCTCAGAATCCAGCGAGAGGCGTGGAGA

HSVd-gJF50D CTTACCTGAGAAAGGAGCCCCGGGGCAACTCTTCTCAGAATCCAGCGAGAGGCGTGGAGA

HSVd-gJF58A CTTACCTGAGAAAGGAGCCCCGGGGCAACTCTTCTCAGAATCCAGCGAGAGGCGTGGAGA

HSVd-g5BB CTTACCTGAGAAAGGAGCCCCGGGGCAACTCTTCTCAGAATCCAGCGAGAGGCGTGGAGA

HSVd-gJF26B CTTACCTGAGAAAGGAGCCCCGGGGCAACTCTTCTCAGAATCCAGCGAGAGGCGTGGAGA

HSVd-gRiesling CTTACCTGAGAAAGGAGCCCCGGGGCAACTCTTCTCAGAATCCAGCGAGAGGCGTGGAGA

HSVd-gJF49B CTTACCTGAGAAAGGAGCCCCGGGGCAACTCTTCTCAGAATCCAGCGAGAGGCGTGGAGA

HSVd-gJF42B CTTACCTGAGAAAGGAGCCCCGGGGCAACTCTTCTCAGAATCCAGCGAGAGGCGTGGAGA

HSVd-gJF26A CTTACCTGAGAAAGGAGCCCCGGGGCAACTCTTCTCAGAATCCAGCGAGAGGCGTGGAGA

HSVd-gJF42A CTTACCTGAGAAAGGAGCCCCGGGGCAACTCTTCTCAGAATCCAGCGAGAGGCGTGGAGA

HSVd-gJF49A CTTACCTGAGAAAGGAGCCCCGGGGCAACTCTTCTCAGAATCCAGCGAGAGGCGTGGAGA

HSVd-gJF64 CTTACCTGAGAAAGGAGCCCCGGGGCAACTCTTCTCAGAATCCAGCGAGAGGCGTGGAGA

HSVd-gJF65C CTTACCTGAGAAAGGAGCCCCGGGGCAACTCTTCTCAGAATCCAGCGAGAGGCGTGGAGA

HSVd-gJF72 CTTACCTGAGAAAGGAGCCCCGGGGCAACTCTTCTCAGAATCCAGCGAGAGGCGTGGAGA

HSVd-gJF58G CTTACCTGAGAAAGGAGCCCCGGGGCAACTCTTCTCAGAATCCAGCGAGAGGCGTGGAGA

***************************************************** ******

HSVd-gJF10 GAGGGCCGCGGTGCTCTGGAGTAGAGGCTCTGCCTTCGAAACACCATCGATCGTCCCTTC

HSVd-gJF58B GAGGGCCGCGGTGCTCTGGAGTAGAGGCTCTGCCTTCGAAACACCATCGATCGTCCCTTC

HSVd-gJF50C GAGGGCCGCGGTGCTCTGGAGTAGAGGCTCTGCCTTCGAAACACCATCGATCGTCCCTTC

HSVd-gJF52 GAGGGCCGCGGTGCTCTGGAGTAGAGGCTCTGCCTTCGAAACACCATCGATCGTCCCTTC

HSVd-gJF65A GAGGGCCGCGGTGCTCTGGAGTAGAGGCTCTGCCTTCGAAACACCATCGATCGTCCCTTC

HSVd-gJAUrbana GAGGGCCGCGGTGCTCTGGAGTAGAGGCTCTGCCTTCGAAACACCATCGATCGTCCCTTC

HSVd-gJF4 GAGGGCCGCGGTGCTCTGGAGTAGAGGCTCTGCCTTCGAAACACCATCGATCGTCCCTTC

HSVd-gJF5 GAGGGCCGCGGTGCTCTGGAGTAGAGGCTCTGCCTTCGAAACACCATCGATCGTCCCTTC

HSVd-gJF7 GAGGGCCGCGGTGCTCTGGAGTAGAGGCTCTGCCTTCGAAACACCATCGATCGTCCCTTC

HSVd-gJF13 GAGGGCCGCGGTGCTCTGGAGTAGAGGCTCTGCCTTCGAAACACCATCGATCGTCCCTTC

HSVd-gJF19 GAGGGCCGCGGTGCTCTGGAGTAGAGGCTCTGCCTTCGAAACACCATCGATCGTCCCTTC

HSVd-gJF25 GAGGGCCGCGGTGCTCTGGAGTAGAGGCTCTGCCTTCGAAACACCATCGATCGTCCCTTC

HSVd-gJF26 GAGGGCCGCGGTGCTCTGGAGTAGAGGCTCTGCCTTCGAAACACCATCGATCGTCCCTTC

HSVd-gJF28 GAGGGCCGCGGTGCTCTGGAGTAGAGGCTCTGCCTTCGAAACACCATCGATCGTCCCTTC

HSVd-gJF29 GAGGGCCGCGGTGCTCTGGAGTAGAGGCTCTGCCTTCGAAACACCATCGATCGTCCCTTC

HSVd-gJA92 GAGGGCCGCGGTGCTCTGGAGTAGAGGCTCTGCCTTCGAAACACCATCGATCGTCCCTTC

HSVd-gJF58D GAGGGCCGCGGTGCTCTGGAGTAGAGGCTCTGCCTTCGAAACACCATCGATCGTCCCTTC

HSVd-gJF58C GAGGGCCGCGGTGCTCTGGAGTAGAGGCTCTGCCTTCGAAACACCATCGATCGTCCCTTC

HSVd-gJF50A GAGGGCCGCGGTGCTCTGGAGTAGAGGCTCTGCCTTCGAAACACCATCGATCGTCCCTTC

HSVd-gJF57 GAGGGCCGCGGTGCTCTGGAGTAGAGGCTCTGCCTTCGAAACACCATCGATCGTCCCTTC

HSVd-gJF65B GAGGGCCGCGGTGCTCTGGAGTAGAGGCTCTGCCTTCGAAACACCATCGATCGTCCCTTC

HSVd-gJF50B GAGGGCCGCGGTGCTCTGGAGTAGAGGCTCTGCCTTCGAAACACCATCGATCGTCCCTTC

HSVd-gJF58F GAGGGCCGCGGTGCTCTGGAGTAGAGGCTCTGCCTTCGAAACACCATCGATCGTCCCTTC

HSVd-gJF58E GAGGGCCGCGGTGCTCTGGAGTAGAGGCTCTGCCTTCGAAACACCATCGATCGTCCCTTC

HSVd-gJF50D GAGGGCCGCGGTGCTCTGGAGTAGAGGCTCTGCCTTCGAAACACCATCGATCGTCCCTTC

HSVd-gJF58A GAGGGCCGCGGTGCTCTGGAGTAGAGGCTCTGCCTTCGAAACACCATCGATCGTCCCTTC

HSVd-g5BB GAGGGCCGCGGTGCTCTGGAGTAGAGGCTCTGCCTTCGAAACACCATCGATCGTCCCTTC

HSVd-gJF26B GAGGGCCGCGGTGCTCTGGAGTAGAGGCTCTGCCTTCGAAACACCATCGATCGTCCCTTC

HSVd-gRiesling GAGGGCCGCGGTGCTCTGGAGTAGAGGCTCTGCCTTCGAAACACCATCGATCGTCCCTTC

HSVd-gJF49B GAGGGCCGCGGTGCTCTGGAGTAGAGGCTCTGCCTTCGAAACACCATCGATCGTCCCTTC

HSVd-gJF42B GAGGGCCGCGGTGCTCTGGAGTAGAGGCTCTGCCTTCGAAACACCATCGATCGTCCCTTC

HSVd-gJF26A GAGGGCCGCGGTGCTCTGGAGTAGAGGCTCTGCCTTCGAAACACCATCGATCGTCCCTTC

HSVd-gJF42A GAGGGCCGCGGTGCTCTGGAGTAGAGGCTCTGCCTTCGAAACACCATCGATCGTCCCTTC

HSVd-gJF49A GAGGGCCGCGGTGCTCTGGAGTAGAGGCTCTGCCTTCGAAACACCATCGATCGTCCCTTC

HSVd-gJF64 GAGGGCCGCGGTGCTCTGGAGTAGAGGCTCTGCCTTCGAAACACCATCGATCGTCCCTTC

HSVd-gJF65C GAGGGCCGCGGTGCTCTGGAGTAGAGGCTCTGCCTTCGAAACACCATCGATCGTCCCTTC

HSVd-gJF72 GAGGGCCGCGGTGCTCTGGAGTAGAGGCTCTGCCTTCGAAACACCATCGATCGTCCCTTC

HSVd-gJF58G GAGGGCCGCGGTGCTCTGGAGTAGAGGCTCTGCCTTCGAAACACCATCGATCGTCCCTTC

************************************************************

HSVd-gJF10 TTCTTTACCTTCTTCTGGCTCTTCCGATGAGACGCGACCGGTGGCATCACCTCTCGGTTC

HSVd-gJF58B TTCTTTACCTTCTTCTGGCTCT-CCGATGAGACGCGACCGGTGGCATCACCTCTCGGTTC

HSVd-gJF50C TTCTTTACCTTCTTCTGGCTCTTCCGATGAGACGCGACCGGTGGCATCACCTCTCGGTTC

HSVd-gJF52 TTCTTTACCTTCTTCTGGCTCTTCCGATGAGACGCGACCGGTGGCATCACCTCTCGGTTC

HSVd-gJF65A TTCTTTACCTTCTTCTGGCTCTTCCGATGAGACGCGACCGGTGGCATCACCTCTCGGTTC

HSVd-gJAUrbana TTCTTTACCTTCTTCTGGCTCTTCCGATGAGACGCGACCGGTGGCATCACCTCTCGGTTC

HSVd-gJF4 TTCTTTACCTTCTTCTGGCTCTTCCGATGAGACGCGACCGGTGGCATCACCTCTCGGTTC

HSVd-gJF5 TTCTTTACCTTCTTCTGGCTCTTCCGATGAGACGCGACCGGTGGCATCACCTCTCGGTTC

HSVd-gJF7 TTCTTTACCTTCTTCTGGCTCTTCCGATGAGACGCGACCGGTGGCATCACCTCTCGGTTC

HSVd-gJF13 TTCTTTACCTTCTTCTGGCTCTTCCGATGAGACGCGACCGGTGGCATCACCTCTCGGTTC

HSVd-gJF19 TTCTTTACCTTCTTCTGGCTCTTCCGATGAGACGCGACCGGTGGCATCACCTCTCGGTTC

HSVd-gJF25 TTCTTTACCTTCTTCTGGCTCTTCCGATGAGACGCGACCGGTGGCATCACCTCTCGGTTC

HSVd-gJF26 TTCTTTACCTTCTTCTGGCTCTTCCGATGAGACGCGACCGGTGGCATCACCTCTCGGTTC

HSVd-gJF28 TTCTTTACCTTCTTCTGGCTCTTCCGATGAGACGCGACCGGTGGCATCACCTCTCGGTTC

HSVd-gJF29 TTCTTTACCTTCTTCTGGCTCTTCCGATGAGACGCGACCGGTGGCATCACCTCTCGGTTC

HSVd-gJA92 TTCTTTACCTTCTTCTGGCTCTTCCGATGAGACGCGACCGGTGGCATCACCTCTCGGTTC

HSVd-gJF58D TTCTTTACCTTCTTCTGGCTCTTCCGATGAGACGCGACCGGTGGCATCACCTCTCGGTTC

HSVd-gJF58C TTCTTTACCTTCTTCTGGCTCTTCCGATGAGACGCGACCGGTGGCATCACCTCTCGGTTC

HSVd-gJF50A TTCTTTACCTTCTTCTGGCTCTTCCGATGAGACGCGACCGGTGGCATCACCTCTCGGTTC

HSVd-gJF57 TTCTTTACCTTCTTCTGGCTCTTCCGATGAGACGCGACCGGTGGCATCACCTCTCGGTTC

HSVd-gJF65B TTCTTTACCTTCTTCTGGCTCTTCCGATGAGACGCGACCGGTGGCATCACCTCTCGGTTC

HSVd-gJF50B ATCTTTACCTTCTTCTGGCTCTTCCGATGAGACGCGACCGGTGGCATCACCTCTCGGTTC

HSVd-gJF58F ATCTTTACCTTCTTCTGGCTCTTCCGATGGGACGCGACCGGTGGCATCACCTCTCGGTTC

HSVd-gJF58E ATCTTTACCTTCTTCTGGCTCTTCCGATGAGACGCGACCGGTGGCATCACCTCTCGGTTC

HSVd-gJF50D ATCTTTACCTTCTTCTGGCTCTTCCGATGAGACGCGACCGGTGGCATCACCTCTCGGTTC

HSVd-gJF58A TTCTTTACCTTCTTCTGGCTCTTCCGATGAGACGCGACCGGTGGCATCACCTCTCGGTTC

HSVd-g5BB TTCTTTACCTTCTTCTGGCTCTTCCGATGAGACGCGACCGGTGGCATCACCTCTCGGTTC

HSVd-gJF26B TTCTTTACCTTCTTCTGGCTCTTCCGATGAGACGCGACCGGTGGCATCACCTCTCGGTTC

HSVd-gRiesling TTCTTTACCTTCTTCTGGCTCTTCCGATGAGACGCGACCGGTGGCATCACCTCTCGGTTC

HSVd-gJF49B TTCTTTACCTTCTTCTGGCTCTTCCGATGAGACGCGACCGGTGGCATCACCTCTCGGTTC

HSVd-gJF42B TTCTTTACCTTCTTCTGGCTCTTCCGATGAGACGCGACCGGTGGCATCACCTCTCGGTTC

HSVd-gJF26A TTCTTTACCTTCTTCTGGCTCTTCCGATGAGACGCGACCGGTGGCATCACCTCTCGGTTC

HSVd-gJF42A TTCTTTACCTTCTTCTGGCTCTTCCGATGAGACGCGACCGGTGGCATCACCTCTCGGTTC

HSVd-gJF49A TTCTTTACCTTCTTCTGGCTCTTCCGATGAGACGCGACCGGTGGCATCACCTCTCGGTTC

HSVd-gJF64 TTCTTTACCTTCTTCTGGCTCTTCCGATGAGACGCGACCGGTGGCATCACCTCTCGGTTC

HSVd-gJF65C TTCTTTACCTTCTTCTGGCTCTTCCGATGAGACGCGACCGGTGGCATCACCTCTCGGTTC

HSVd-gJF72 TTCTTTACCTTCTTCTGGCTCTTCCGATGAGACGCGACCGGTGGCATCACCTCTCGGTTC

HSVd-gJF58G TTCTTTACCTTCTTCTGGCTCTTCCGATGAGACGCGACCGGTGGCATCACCTCTCGGTTC

********************* ****** ******************************

**Nucleotide position 180**

HSVd-gJF10 GTCCCAACCTGCTTTTTGTCTATCTGAGCCTCTGCCGCGGATCCTCTCTTGAGCCCCT

HSVd-gJF58B GTCCCAACCTGCTTTTTGTCTATCTGAGCCTCTGCCGCGGATCCTCTCTTGAGCCCCT

HSVd-gJF50C GTCCCAACCTGCTTTTTGTCTATCTGAGCCTCTGCCGCGGATCCTCTCTTGAGCCCCT

HSVd-gJF52 GTCCCAACCTGCTTTTTGTCTATCTGAGCCTCTGCCGCGGATCCTCTCTTGAGCCCCT

HSVd-gJF65A GTCCCAACCTGCTTTTTGTCTATCTGAGCCTCTGCCGCGGATCCTCTCTTGAGCCCCT

HSVd-gJAUrbana GTCCCAACCTGCTTTTTGTCTATCTGAGCCTCTGCCGCGGATCCTCTCTTGAGCCCCT

HSVd-gJF4 GTCCCAACCTGCTTTTTGTCTATCTGAGCCTCTGCCGCGGATCCTCTCTTGAGCCCCT

HSVd-gJF5 GTCCCAACCTGCTTTTTGTCTATCTGAGCCTCTGCCGCGGATCCTCTCTTGAGCCCCT

HSVd-gJF7 GTCCCAACCTGCTTTTTGTCTATCTGAGCCTCTGCCGCGGATCCTCTCTTGAGCCCCT

HSVd-gJF13 GTCCCAACCTGCTTTTTGTCTATCTGAGCCTCTGCCGCGGATCCTCTCTTGAGCCCCT

HSVd-gJF19 GTCCCAACCTGCTTTTTGTCTATCTGAGCCTCTGCCGCGGATCCTCTCTTGAGCCCCT

HSVd-gJF25 GTCCCAACCTGCTTTTTGTCTATCTGAGCCTCTGCCGCGGATCCTCTCTTGAGCCCCT

HSVd-gJF26 GTCCCAACCTGCTTTTTGTCTATCTGAGCCTCTGCCGCGGATCCTCTCTTGAGCCCCT

HSVd-gJF28 GTCCCAACCTGCTTTTTGTCTATCTGAGCCTCTGCCGCGGATCCTCTCTTGAGCCCCT

HSVd-gJF29 GTCCCAACCTGCTTTTTGTCTATCTGAGCCTCTGCCGCGGATCCTCTCTTGAGCCCCT

HSVd-gJA92 GTCCCAACCTGCTTTTTGTCTATCTGAGCCTCTGCCGCGGATCCTCTCTTGAGCCCCT

HSVd-gJF58D GTCCCAACCTGCTTTTTGTCTATCTGAGCCCCTGCCGCGGATCCTCTCTTGAGCCCCT

HSVd-gJF58C GTCCCAACCTGCTTTTTGTCTATCTGAGCCTCTGCCGCGGATCCTCTCTTGAGCCCCT

HSVd-gJF50A GTCCCAACCTGCTTTTTGTCTATCTGAGCCTCTGCCGCGGATCCTCTCTTGAGCCCCT

HSVd-gJF57 GTCCCAACCTGCTTTTTGTCTATCTGAGCCTCTGCCGCGGATCCTCTCTTGAGCCCCT

HSVd-gJF65B GTCCCAACCTGCTTTTTGTCTATCTGAGCCTCTGCCGCGGATCCTCTCTTGAGCCCCT

HSVd-gJF50B GTCCCAACCTGCTTTTTGTCTATCTGAGCCTCTGCCGCGGATCCTCTCTTGAGCCCCT

HSVd-gJF58F GTCCCAACCTGCTTTTTGTCTATCTGAGCCTCTGCCGCGGATCCTCTCTTGAGCCCCT

HSVd-gJF58E GTCCCAACCTGCTTTTTGTCTATCTGAGCCTCTGCCGCGGATCCTCTCTTGAGCCCCT

HSVd-gJF50D GTCCCAACCTGCTTTTTGTCTATCTGAGCCTCTGCCGCGGATCCTCTCTTGAGCCCCT

HSVd-gJF58A GTCCCAACCTGCTTTTGTTCTATCTGAGCCTCTGCCGCGGATCCTCTCTTGAGCCCCT

HSVd-g5BB GTCCCAACCTGCTTTTGTTCTATCTGAGCCTCTGCCGCGGATCCTCTCTTGAGCCCCT

HSVd-gJF26B GTCCCAACCTGCTTTTGTTCTATCTGAGCCTCTGCCGCGGATCCTCTCTTGAGCCCCT

HSVd-gRiesling GTCCCAACCTGCTTTTGTTCTATCTGAGCCTCTGCCGCGGATCCTCTCTTGAGCCCCT

HSVd-gJF49B GTCCCAACCTGCTTTTGTTCTATCTGAGCCTCTGCCGCGGATCCTCTCTTGAGCCCCT

HSVd-gJF42B GTCCCAACCTGCTTTTGTTCTATCTGAGCCTCTGCCGCGGATCCTCTCTTGAGCCCCT

HSVd-gJF26A GTCCCAACCTGCTTTTGTTCTATCTGAGCCTCTGCCGCGGATCCTCTCTTGAGCCCCT

HSVd-gJF42A GTCCCAACCTGCTTTTGTTCTATCTGAGCCTCTGCCGCGGATCCTCTCTTGAGCCCCT

HSVd-gJF49A GTCCCAACCTGCTTTTGTTCTATCTGAGCCTCTGCCGCGGATCCTCTCTTGAGCCCCT

HSVd-gJF64 GTCCCAACCTGCTTTTGTTCTATCTGAGCCTCTGCCGCGGATCCTCTCTTGAGCCCCT

HSVd-gJF65C GTCCCAACCTGCTTTTGTTCTATCTGAGCCTCTGCCGCGGATCCTCTCTTGAGCCCCT

HSVd-gJF72 GTCCCAACCTGCTTTTGTTCTATCTGAGCCTCTGCCGCGGATCCTCTCTTGAGCCCCT

HSVd-gJF58G GTCCCAACCTGCTTTTTGTCTATCTGAGCCTCTGCCGCGGATCCTCTCTTGAGCCCCT

**************** ************ ***************************

**Nucleotide position** **256-257**

* Nucleotide with yellow background showed major mutation positions. Those with red background showed singleton mutations which we did not consider as major or minor mutation position in the text.
