# Supplementary material for: Cultivated Grapevines Represent a Symptomless Reservoir for the Transmission of Hop Stunt Viroid to Hop Crops: 15 Years of Evolutionary Analysis
Source: PLoS One. 2009 Dec 24;4(12):e8386. doi: 10.1371/journal.pone.0008386 (PMC2793511; doi:10.1371/journal.pone.0008386)
Supplement: Table S2 — Multiple sequence alignment of HpSVd-grapevine deposited in SubViral RNA database (0.07 MB DOC) [file pone.0008386.s004.doc]

**Multiple sequence alignment of HpSVd-grapevine deposited in SubViral RNA database**

**Name Sequence**

HSVd-g5BB CTGGGGAATTCTCGAGTTGCCGCATCAGGCATGCAAAGAAAAAAACTTCGCAGGGA-GGT

HSVd.gv003 CTGGGGAATTCTCGAGTTGCCGCATCAGGCATGCAAAGAAAAAAACTTCGCAGGGA-GGT

HSVd-gRiesling CTGGGGAATTCTCGAGTTGCCGCATAAGGCATGCAAAGAAAAAAACTTGGCAGGGA-GGT

HSVd.gv009 CTGGGGAATTCTCGAGTTGCCGCATAAGGCATGCAAAGAAAAAAACTTGGCAGGGA-GGT

HSVd.gv002 CTGGGGAATTCTCGAGTTGCCGCATAAGGCATGCAAAGAAAAAAACTTGGCAGGGA-GGT

HSVd-grape CTGGGGAATTCTCGAGTTGCCGCATCAGGCAAGCAAAGAAAA-AACAAGGCAGGGA-GGT

HSVd.gv061 CTGGGGAATTCTCGAGTTGCCGCATCAGGCAAGCAAAGAAAA-AACAAGGCAGGGA-GGT

HSVd.gv042 CTGGGGAATTCTCGAGTTGCCGCATCAGGCAAGCAAAGAAAA-AACAAGGCAGGGA-GGT

HSVd.gv051 CTGGGGAATTCTCGAGTTGCCGCATCAGGCAAGCAAAGAAAA-AACAAGGCAGGGA-GGT

HSVd.gv059 CTGGGGAATTCTCGAGTTGCCGCATCAGGCAAGCAAAGAAAA-AACAAGGCAGGGA-GGT

HSVd.gv039 CTGGGGAATTCTCGAGTTGCCGCATCAGGCAAGCAAAGAAAA-AACAAGGCAGGGA-GGT

HSVd.gv047 CTGGGGAATTCTCGAGTTGCCGCATCAGGCAAGCAAAGAAAA-AACAAGGCAGGGA-GGT

HSVd.gv056 CTGGGGAATTCTCGAGTTGCCGCATCAGGCAAGCAAAGAAAA-AACAAGGCAGGGA-GGT

HSVd.gv058 CTGGGGAATTCTCGAGTTGCCGCATCAGGCAAGCAAAGAAAA-AACAAGGCAGGGA-GGT

HSVd.gv001 CTGGGGAATTCTCGAGTTGCCGCATCAGGCAAGCAAAGAAAA-AACAAGGCAGGGA-GGT

HSVd.gv007 CTGGGGAATTCTCGAGTTGCCGCATCAGGCAAGCAAAGAAAA-AACAAGGCAGGGA-GGT

HSVd.gv035 CTGGGGAATTCTCGAGTTGCCGCATCAGGCAAGCAAAGAAAA-AACAAGGCAGGGA-GGT

HSVd.gv045 CTGGGGAATTCTCGAGTTGCCGCATCAGGCAAGCAAAGAAAA-AACAAGGCAGGGA-GGT

HSVd.gv054 CTGGGGAATTCTCGAGTTGCCGCATCAGGCAAGCAAAGAAAA-AACAAGGCAGGGA-GGT

HSVd.gv008 CTGGGGAATTCTCGAGTTGCCGCATCAGGCAAGCAAAGAAAA-AACAAGGCAGGGA-GGT

HSVd.gv040 CTGGGGAATTCTCGAGTTGCCGCATCAGGCAAGCAAAGAAAA-AACAAGGCAGGGA-GGT

HSVd.gv052 CTGGGGAATTCTCGAGTTGCCGCATCATGCAAGCAAAGAAAA-AACAAGGCAGGGA-GGT

HSVd.gv036 CTGGGGAATTCTCGAGTTGCCGCATCAGGCAAGCAAAGAAAA-AACAAGGCAGGGA-GGT

HSVd.gv055 CTGGGGAATTCTCGAGTTGCCGCATCAGGCAAGCAAAGAAAA-AACAAGGCAGGGA-GGT

HSVd.gv005 CTGGGGAATTCTCGAGTTGCCGCATCAGGCAAGCAAAGAAA--AACAAGGCAGGGA-GGT

HSVd.gv037 CTGGGGAATTCTCGAGTTGCCGCATCAGGCAAGCAAAGAAAG-AACAAGGCAGGGA-GGT

HSVd.gv027 CTGGGGAATTCTCGAGTTGCCGCATCAGGCAAGCAAAGAAAA-AACAAGGCAGGGA-GGT

HSVd.gv032 CTGGGGAATTCTCGAGTTGCCGCATCAGGCAAGCAAAGAAAA-AACAAGGCAGGGA-GGT

HSVd.gv043 CTGGGGAATTCTCGAGTTGCCGCATCAGGCAAGCAAAGAAAA-AACAAGGCAGGGA-GGT

HSVd.gv053 CTGGGGAATTCTCGAGTTGCCGCATCAGGCAAGCAAAGAAAA-AACAAGGCAGGGA-GGT

HSVd.gv062 CTGGGGAATTCTCGAGTTGCCGCATCAGGCAAGCAAAGAAAA-AACAAGGCAGGGA-GGT

HSVd.gv044 CTGGGGAATTCTCGAGTTGCCGCATCAGGCAAGCAAAGAAAA-AACAAGGCAGGGA-GGT

HSVd.gv049 CTGGGGAATTCTCGAGTTGCCGCATCAGGCAAGCAAAGAAAA-AACAAGGCAGGGA-GGT

HSVd.gv004 CTGGGGAATTCTCGAGTTGCCGCATCAGGCAAGCAAAGAAAA-AACAAGGCAGGGA-GGT

HSVd.gv006 CTGGGGAATTCTCGAGTTGCCGCATCAGGCAAGCAAAGAAAA-AACAAGGCAGGGA-GGT

HSVd.gv046 CTGGGGAATTCTCGAGTTGCCGCATCAGGCAAGCAAAGAAAA-AACAAGGCAGGGA-GGT

HSVd.gv011 CTGGGGAATTCTCGAGTTGCCGCATCAGGCAAGCAAAGAAAA-AACAAGGCAGGGA-GGT

HSVd.gv060 CTGGGGAATTCTCGAGTTGCCGCATCAGGCAAGCAAAGAAAA-AACAAGGCAGGGA-GGT

HSVd.gv048 CTGGGGAATTCTCGAGTTGCCGCATCAGGCAAGCAAAGAAAA-AACAAGGCAGGGA-GGT

HSVd.gv041 CTGGGGAATTCTCGAGTTGCCGCATCAGGCAAGCAAAGAAAA-AACAAGGCAGGGA-GGT

HSVd.gv029 CTGGGGAATTCTCGAGTTGCCGCATCAGGCAAGCAAAGAAAA-AACAAGGCAGGGA-GGT

HSVd.gv031 CTGGGGAATTCTCGAGTTGCCGCATCAGGCAAGCAAAGAAAA-AACAAGGCAGGGA-GGT

HSVd.gv034 CTGGGGAATTCTCGAGTTGCCGCATCAGGCAAGCAAAGAAAA-AACAAGGCAGGGA-GGT

HSVd.gv030 CTGGGGAATTCTCGAGTTGCCGCATCAGGCAAGCAAAGAAAA-AACAAGGCAGGGA-GGT

HSVd.gv033 CTGGGGAATTCTCGAGTTGCCGCATCAGGCAAGCAAAGAAAA-AACAAGGCAGGGA-GGT

HSVd.gv057 CTGGGGAATTCTCGAGTTGCCGCATCAGGCAAGCAAAGAAAA-AACAAGGCAGGGA-GGT

HSVd.gv038 CTGGGGAATTCTCGAGTTGCCGCAAAAGGCATGCAAAGAAAAAAACTAGGCAGGGA-GGC

HSVd.gv050 CTGGGGAATTCTCGAGTTGCCGCAAAAGGCATGCAAAGAAAAAAACTAGGCAGGGAAGGT

************************ * *** ********* *** ******* **

**Nucleotide position 26 32 44/45, 46-47**

HSVd-g5BB ACTTACCTGAGAAAGGAGCCCCGGGGCAACT-CTTCTCAGAATCCAGCGAGAGGCGT-GG

HSVd.gv003 ACTTACCTGAGAAAGGAGCCCCGGGGCAACT-CTTCTCAGAATCCAGCGAGAGGCGT-GG

HSVd-gRiesling ACTTACCTGAGAAAGGAGCCCCGGGGCAACT-CTTCTCAGAATCCAGCGAGAGGCGT-GG

HSVd.gv009 ACTTACCTGAGAAAGGAGCCCCGGGGCAACT-CTTCTCAGAATCCAGCGAGAGGCAT-GG

HSVd.gv002 ACTTACCTGAGAAAGGAGCCCCGGGGCAACT-CTTCTCAGAATCCAGCGAGAGGCGT-GG

HSVd-grape ACTTACCTGAGAAAGGAGCCCCGGGGCAACT-CTTCTCAGAATCCAGCGAGAGGCGT-GG

HSVd.gv061 ACTTACCTGAGAAAGGAGCCCCGGGGCAACT-CT-CTCAGAATCCAGCGAGAGGCGT-GG

HSVd.gv042 ACTTACCTGAGAAAGGAGCCCCGGGGCAACT-CTTCTCAGAATCCAGCGAGAGGCGT-GG

HSVd.gv051 ACTTACCTGAGAAAGGAGCCCCGGGGCAACT-CTTCTCAGAATCCAGCGAGAGGCGT-GG

HSVd.gv059 ACTTACCTGAGAAAGGAGCCCCGGGGCAACT-CTTCTCAGAATCCAGCGAGAGGCGT-GG

HSVd.gv039 ACTTACCTGAGAAAGGAGCCCCGGGGCAACT-CTTCTCAGAATCCAGCGAGAGGCGT-GG

HSVd.gv047 ATTTACCTGAGAAAGGAGCCCCGGGGCAACT-CTTCTCAGAATCCAGCGAGAGGCGT-GG

HSVd.gv056 ACTTACCTGAGAAAGGAGCCCCGGGGCAACT-CTTCTCAGAATCCAGCGGGAGGCGT-GG

HSVd.gv058 ACTTACCTGAGAAAGGAGCCCCGGGGCAACT-CTTCTCAGAATCCAGCGGGAGGCGT-GG

HSVd.gv001 ACTTACCTGAGAAAGGAGCCCCGGGGCAACT-CTTCTCAGAATCCAGCGAGAGGCGT-GG

HSVd.gv007 ACTTACCTGAGAAAGGAGCCCCGGGGCAACT-CTTCTCAGAATCCAGCGAGAGGCGT-GG

HSVd.gv035 ACTTACCTGAGAAAGGAGCCCCGGGGCAACT-CTTCTCAGAATCCAGCGAGAGGCGT-GG

HSVd.gv045 ACTTACCTGAGAAAGGAGCCCCGGGGCAACT-CTTCTCAGAATCCAGCGAGAGGCGT-GG

HSVd.gv054 ACTTACCTGAGAAAGGAGCCCCGGGGCAACT-CTTCTCAGAATCCAGCGAGAGGCGT-GG

HSVd.gv008 ACTTACCTGAGAAAGGAGCCCCGGGGCAACT-CTTCTCAGAATCCAGCGAGAGGCGT-GG

HSVd.gv040 ACTTACCTGAGAAAGGAGCCACGGGGCAACT-CTTCTCAGAATCCAGCGAGAGGCGT-GG

HSVd.gv052 ACTTACCTGAGAAAGGAGCCCCGGGGCAACT-CTTCTCAGAATCCAGCGAGAGGCGT-GG

HSVd.gv036 ACTTACCTGAGAAAGGAGCCCCGGGGCAACT-CTTCTCAGAATCCAGCGAGAGGCGT-GG

HSVd.gv055 ACTTACCTGAGAAAGGAGCCCCGGGGCAACT-CTTCTCAGAATCCAGCGAGAGGCGT-GG

HSVd.gv005 ACTTACCTGAGAAAGGAGCCCCGGGGCAACT-CTTCTCAGAATCCAGCGAGAGGCGT-GG

HSVd.gv037 ACTTACCTGAGAAAGGAGCCCCGGGGCAACT-CTTCTCAGAATCCAGCGAGAGGCGT-GG

HSVd.gv027 ACTTACCTGAGAAAGGAGCCCCGGGGCAACTTCTTCTCAGAATCCAGCGAGAGGCGT-GG

HSVd.gv032 ACTTACCTGAGAAAGGAGCCCCGGGGCAACT-CTTCTCAGAATCCAGCGAGAGGCGT-GG

HSVd.gv043 ACTTACCTGAGAAAGGAGCCCCGGGGCAACT-CTTCTCAGAATCCAGCGAGAGGCGT-GG

HSVd.gv053 ACTTACCTGAGAAAGGAGCCCCGGGGCAACT-CTTCTCAGAATCCAGCGAGAGGCGT-GG

HSVd.gv062 ACTTACCTGAGAAAGGAGCCCCGGGGCAACT-CTTCTCAGAATCCAGCGAGAGGCGT-GG

HSVd.gv044 ACTTACCTGAGAAAGGAGCCCCGGGGCAACT-CTTCTCAGAATCCAGCGAGAGGCGT-GG

HSVd.gv049 ACTTACCTGAGAAAGGAGCCCCGGGGCAACT-CTTCTCAGAATCCAGCGAGAGGCGT-GG

HSVd.gv004 ACTTACCTGAGAAAGGAGCCCCGGGGCAACT-CTTCTCAGAATCCAGCGAGAGACGT-GG

HSVd.gv006 ACTTACCTGAGAAAGGAGCCCCGGGGCAACT-CTTCTCAGAATCCAGCGAGAGACGT-GG

HSVd.gv046 ACTTACCTGAGAAAGGAGCCCCGGGGCAACT-CTTCTCAGAATCCAGCGAGAGGCGT-GG

HSVd.gv011 ACTTACCTGAGAAAGGAGCCCCGGGGCAACT-CTTCTCAGAATCCAGCGAGAGGCGT-GG

HSVd.gv060 ACTTACCTGAGAAAGGAGCCCCGGGGCAACT-CTTCTCAGAATCCAGCGAGAGGCGT-GG

HSVd.gv048 ACTTACCTGAGAAAGGAGCCCCGGGGCAACT-CTTCTCAGAATCCAGCGAGAGGCGT-GG

HSVd.gv041 ACTTACCTGAGAAAGGAGCCCCGGGGCAACT-CTTCTCAGAATCCAGCGAGAGGCGT-GG

HSVd.gv029 ACTTACCTGAGAAAGGAGCCCCGGGGCAACT-CTTCTCAGAATCCAGTGAGAGGCGT-GG

HSVd.gv031 ACTTACCTGAGAAAGGAGCCCCGGGGCAACT-CTTCTCAGAATCCAGTGAGAGGCGT-GG

HSVd.gv034 ACTTACCTGAGAAAGGAGCCCCGGGGCAACT-CTTCTCAGAATCCAGTGAGAGGCGT-GG

HSVd.gv030 ACTTACCTGAGAAAGGAGCCCCGGGGCAACT-CTTCTCAGAATCCAGCGAGAGGCGTAGG

HSVd.gv033 ACTTACCTGAGAAAGGAGCCCCGGGGCAACT-CTTCTCAGAATCCAGCGAGAGGCGT-GG

HSVd.gv057 ACTTACCTGAGAAAGGAGCCCCGGGGCAACT-CTTCTCAGAATCCAGCGAGAGGCGT-GG

HSVd.gv038 GCTTACCTGAGAAAGGAGCCCCGGGGCAACT-CTTCTCAGAATCCAGCGAGAGGCGT-GG

HSVd.gv050 GCTTACCTGAGAAAGGAGCCCCGGGGCAACT-CTTCTCAGAATCCAGCGAGAGGCGT-GG

****************** ********** ** ************ * *** * * **

**nucleotide position 105**

HSVd-g5BB AGAGAGGGCCGCGGTGCTCTGGAGTAGAGGCT-CTGCCTTC-GAAACACCATCGATCGTC

HSVd.gv003 AGAGAGGGCCGCGGTGCTCTGGAGTAGAGGCT-CTGCCTTC-GAAACACCATCGATCGTC

HSVd-gRiesling AGAGAGGGCCGCGGTGCTCTGGAGTAGAGGCT-CTGCCTTC-GAAACACCATCGATCGTC

HSVd.gv009 AGAGAGGGCCGCGGTGCTCTGGAGTAGAGGCT-CTGCCTTC-GAAACACCATCGATCGTC

HSVd.gv002 AGAGAGGGCCGCGGTGCTCTGGAGTAGAGGCT-CTGCCTTC-GAAACACCATCGATCGTC

HSVd-grape AGAGAGGGCCGCGGTGCTCTGGAGTAGAGGCT-CTGCCTTC-GAAACACCATCGATCGTC

HSVd.gv061 AGAGAGGGCCGCGGTGCCCTGGAGTAGAGGCT-CTGCCTTC-GAAACACCATCGATCGTC

HSVd.gv042 AGAGAGGGCCGCGGTGCTCTGGAGTAGAGGCT-CTGCCTTC-GAAACACCATCGATCGTC

HSVd.gv051 AGAGAGGGCCGCGGTGCTCTGGAGTAGAGGCT-CTGCCTTC-GAAACACCATCGATCGTC

HSVd.gv059 AGAGAGGGCCGCGGTGCTCTGGAGTAGAGGCT-CTGCCTTC-GAAACACCATCGATCGTC

HSVd.gv039 AGAGAGGGCCGCGGTGCTCTGGAGTAGAGGCT-CTGCCTTC-GAAACACCATCGATCGTC

HSVd.gv047 AGAGAGGGCCGCGGTGCTCTGGAGTAGAGGCT-CTGCCTTC-GAAACACCATCGATCGTC

HSVd.gv056 AGAGAGGGCCGCGGTGCTCTGGAGTAGAGGCT-CTGCCTTC-GAAACACCATCGATCGTC

HSVd.gv058 AGAGAGGGCCGCGGTGCTCTGGAGTAGAGGCT-CTGCCTTC-GAAACACCATCGATCGTC

HSVd.gv001 AGAGAGGGCCGCGGTGCTCTGGAGTAGAGGCT-CTGCTTC--AGAACACCATCGATCGTC

HSVd.gv007 AGAGAGGGCCGCGGTGCTCTGGAGTAGAGGCT-CTGCCTTCGAAAACACCATCGATCGTC

HSVd.gv035 AGAGAGGGCCGCGGTGCTCTGGAGTAGAGGCT-CTGCCTTC-GAAACACCATCGATCGTC

HSVd.gv045 AGAGAGGGCCGCGGTGCTCTGGAGTAGAGGCT-CTGCCTTC-GAAACACCATCGATCGTC

HSVd.gv054 AGAGAGGGCCGCGGTGCTCTGGAGTAGAGGCT-CTGCCTTC-GAAACACCATCGATCGTC

HSVd.gv008 AGAGAGGGCCGCGGTGCTCTGGAGTAGAGGCT-CTGCCTTC-GAAACACCATCGATCGTC

HSVd.gv040 AGAGAGGGCCGCGGTGCTCTGGAGTAGAGGCT-CTGCCTTC-GAAACACCATCGATCGTC

HSVd.gv052 AGAGAGGGCCGCGGTGCTCTGGAGTAGAGGCT-CTGCCTTC-GAAACACCATCGATCGTC

HSVd.gv036 AGAGAGGGCCGCGGTGCTCTGGAGTAGAGGCT-CTGCCTTC-GAAACACCATCGATCGTC

HSVd.gv055 AGAGAGGGCCGCGGTGCTCTGGAGTAGAGGCT-CTGCCTTC-GAAACACCATCGATCGTC

HSVd.gv005 AGAGAGGGCCGCGGTGCTCTGGAGTAGAGGCT-CTGCCTTC-GAAACACCATCGATCGTC

HSVd.gv037 AGAGAGGGCCGCGGTGCTCTGGAGTAGAGGCT-CTGCCTTC-GAAACACCATCGATCGTC

HSVd.gv027 AGAGAGGGCCGCGGTGCTCTGGAGTAGAGGCT-CTGCCTTC-GAAACACCATCGATCGTC

HSVd.gv032 AGAGAGGGCCGCGGTGCTCTGGAGTAGAGGCT-CTGCCTTC-GAAACACCATCGATCGTC

HSVd.gv043 AGAGAGGGCCGCGGTGCTCTGGAGTAGAGGCT-CTGCCTTC-GAAACACCATCGATCGTC

HSVd.gv053 AGAGAGGGCCGCGGTGCTCTGGAGTAGAGGCT-CTGCCTTC-GAAACACCATCGATCGTC

HSVd.gv062 AGAGAGGGCCGCGGTGCTCTGGAGTAGAGGCT-CTGCCTTC-GAAACACCATCGATCGTC

HSVd.gv044 AGAGAGGGCCGCGGTGCTCTGGAGTAGAGGCT-CTGCCTTC-GAAACACCATCGATCGTC

HSVd.gv049 AGAGAGGGCCGCGGTGCTCTGGAGTAGAGGCT-CTGCCTTC-GAAACACCATCGATCGTC

HSVd.gv004 AGAGAGGGCCGCGGTGCTCTGGAGTAGAGGCT-CTGCCTTC-GAAACACCATCGATCGTC

HSVd.gv006 AGAGAGGGCCGCGGTGCTCTGGAGTAGAGGCT-CTGCCTTC-GAAACACCATCGATCGTC

HSVd.gv046 AGAGAGGGCCGCGGTGCTCTGGAGTAGAGGCT-CTGCCTTC-GAAACACCATCGATCGTC

HSVd.gv011 AGAGAGGGCCGCGGTGCTCTGGAGTAGAGGCT-CTGCCTTC-GAAACACCATCGATCGTC

HSVd.gv060 AGAGAGGGCCGCGGTGCTCTGGAGTAGAGGCT-CTGCCTTC-GAAACACCATCGATCGTC

HSVd.gv048 AGAGAGGGCCGCGGTGCTCTGGAGTAGAGGCT-CTGCCTTC-GAAACACCATCGATCGTC

HSVd.gv041 AGAGAGGGCCGCGGTGCTCTGGAGTAGAGGCT-CTGCCTTC-GAAACACCATCGATCGTC

HSVd.gv029 AGAGAGGGCCGCGGTGCTCTGGAGTAGAGGCT-CTGCCTTC-GAAACACCATCGATCGTC

HSVd.gv031 AGAGAGGGCCGCGGTGCTCTGGAGTAGAGGCT-CTGCCTTC-GAAACACCATCGATCGTC

HSVd.gv034 AGAGAGGGCCGCGGTGCTCTGGAGTAGAGGCT-CTGCCTTC-GAAACACCATCGATCGTC

HSVd.gv030 AGAGAGGGCCGCGGTGCTCTGGAGTAGAGGCTTCTTGCTTC-GAAACACCATCGATCGTC

HSVd.gv033 AGAGAGGGCCGCGGTGCTCTGGAGTAGAGGCT-CTGCCTTC-GAAACACCATCGATCGTC

HSVd.gv057 AGAGAGGGCCGCGGTGCTCTGGAGTAGAGGCT-CTGCCTTC-GAAACACCATCGATCGTC

HSVd.gv038 AGAGAGGGCCGCGGTGCTCTGGAGTAGAGGCT-CTGCCTTC-GAAACACCATCGATCGTC

HSVd.gv050 AGAGAGGGCCGCGGTGCTCTGGAGTAGAGGCT-CTGCCTTC-GAAACACCATCGATCGTC

*****************1**************1**111*11111****************

HSVd-g5BB CCTTCTTCTTT-ACCTTCTTCTGGCTCTTCCGAT-GAGACGCGACCGGTGGCATCACCTC

HSVd.gv003 CCTTCTTCTTT-ACCTTCTTCTGGCTCTTCCGAT-GAGACGCGACCGGTGGCATCACCTC

HSVd-gRiesling CCTTCTTCTTT-ACCTTCTTCTGGCTCTTCCGAT-GAGACGCGACCGGTGGCATCACCTC

HSVd.gv009 CCTTCTTCTTT-ACCTTCTTCTGGCTCTTCCGAT-GAGACGCGACCGGTGGCATCACCTC

HSVd.gv002 CCTTCTTCTTT-ACCTTCTTCTGGCTCTTCCGAT-GAGACGCGACCGGTGGCATCACCTC

HSVd-grape CCTTCTTCTTT-ACCTTCTTCTGGCTCTTCCGAT-GAGACGCGACCGGTGGCATCACCTC

HSVd.gv061 CCTTCTTCTTT-ACCTTCTTCTGGCTCTTCCGAT-GAGACGCGACCGGTGGCATCACCTC

HSVd.gv042 CCTTCTTCTTT-ACCTTCTTCTGGCTCTTCCGAT-GAGACGCGACCGGTGGCATCACCTC

HSVd.gv051 CCTTCTTCTTT-ACCTTCTTCTGGCTCTTCCGAT-GAGACGCGACCGGTGGCATCACCTC

HSVd.gv059 CCTTCTTCTTT-ACCTTCTTCTGGCTCTTCCGAT-GAGACGCGACCGGTGGCATCACCTC

HSVd.gv039 CCTTCTTCTTT-ACCTTCTTCTGGCTCTTCCGAT-GAGACGCGACCGGTGGCATCACCTC

HSVd.gv047 CCTTCTTCTTT-ACCTTCTTCTGGCTCTTCCGAT-GAGACGCGACCGGTGGCATCACCTC

HSVd.gv056 CCTTCTTCTTT-ACCTTCTTCTGGCTCTTCCGAT-GAGACGCGACCGGTGGCATCACCTC

HSVd.gv058 CCTTCTTCTTT-ACCTTCTTCTGGCTCTTCCGAT-GAGACGCGACCGGTGGCATCACCTC

HSVd.gv001 CCTTCTTCTTT-ACCTTCTTCTGGCTCTTCCGAT-GAGACGCGACCGGTGGCATCACCTC

HSVd.gv007 CCTTCTTCTTT-ACCTTCTTCTGGCTCTTCCGAT-GAGACGCGACCGGTGGCATCACCTC

HSVd.gv035 CCTTCTTCTTT-ACCTTCTTCTGGCTCTTCCGAT-GAGACGCGACCGGTGGCATCACCTC

HSVd.gv045 CCTTCTTCTTT-ACCTTCTTCTGGCTCTTCCGAT-GAGACGCGACCGGTGGCATCACCTC

HSVd.gv054 CCTTCTTCTTT-ACCTTCTTCTGGCTCTTCCGAT-GAGACGCGACCGGTGGCATCACCTC

HSVd.gv008 CCTTCTTCTTT-ACCTTCTTCTGGCTCTTCCGAT-GAGACGCGACCGGTGGCATCACCTC

HSVd.gv040 CCTTCTTCTTT-ACCTTCTTCTGGCTCTTCCGAT-GAGACGCGACCGGTGGCATCACCTC

HSVd.gv052 CCTTCTTCTTT-ACCTTCTTCTGGCTCTTCCGAT-GAGACGCGACCGGTGGCATCACCTC

HSVd.gv036 CCTTCTTCTTT-ACCTTCTTCTGGCTCTTCCGAT-GAGACGTGACCGGTGGCATCACCTC

HSVd.gv055 CCTTCTTCTTT-ACCTTCTTCTGGCTCTTCCGAT-GAGACGCGACCGGTGGCATCACCTC

HSVd.gv005 CCTTCTTCTTT-ACCTTCTTCTGGCTCTTCCGAT-GAGACGCGACCGGTGGCATCACCTC

HSVd.gv037 CCTTCTTCTTT-ACCTTCTTCTGGCTCTTCCGAT-GAGACGCGACCGGTGGCATCACCTC

HSVd.gv027 CCTTCTTCTTT-ACCTTCTTCTGGCTCTTCCGAT-GAGACGCGACCGGTGGCATCACCTC

HSVd.gv032 CCTTCTTCTTT-ACCTTCTTCTGGCTCTTCCGAT-GAGACGCGACCGGTGGCATCACCTC

HSVd.gv043 CCTTCTTCTTT-ACCTTCTTCTGGCTCTTCCGAT-GAGACGCGACCGGTGGCATCACCTC

HSVd.gv053 CCTTCTTCTTT-ACCTTCTTCTGGCTCTTCCGAT-GAGACGCGACCGGTGGCATCACCTC

HSVd.gv062 CCTTCTTCTTT-ACCTTCTTCTGGCTCTTCCGAT-GAGACGCGACCGGTGGCATCACCTC

HSVd.gv044 CCTTCTTCTTT-ACCTTCTTCCGGCTCTTCCGAT-GAGACGCGACCGGTGGCATCACCTC

HSVd.gv049 CCTTCTTCTTT-ACCTTCTTCTGGCTCTTCCGAT-GAGACGCGACCGGTGGCATCACCTC

HSVd.gv004 CCTTCTTCTTT-ACCTTCTTCTGGCTCTTCCGAT-GAGACGCGACCGGTGGCATCACCTC

HSVd.gv006 CCTTCTTCTTT-ACCTTCTTCTGGCTCTTCCGAT-GAGACGCGACCGGTGACATCACCTC

HSVd.gv046 CCTTCTTCTTT-ACCTTCTTCTGGCTCTTCCGAT-GAGACGCAACCGGTGGCATCACCTC

HSVd.gv011 CCTTCTTCTTT-ACCTTCTTCTGGCTCTTCCGAT-GAGACGCGACCGGTGGCATCACCTC

HSVd.gv060 CCTTCTTCTTT-ACCTTCTTCTGGCTCTTCCGAT-GAGACGCGACCGGTGGCATCACCTC

HSVd.gv048 CCTTCTTCTTT-ACCTTCTTCTGGCTCTTCCGAT-GAGACGCGACCGGTGGCATCACCTC

HSVd.gv041 CCTTCTTCTTT-TCCTTCTTCTGGCTCTTCCGAT-GAGACGCGACCGGTGGCATCACCTC

HSVd.gv029 CCTTCTTCTTT-ACCTTCTTCTGGCTCTTCTTG--GAGACGCGACCGGTGGCATCACCTC

HSVd.gv031 CCTTCTTCTTT-ACCTTCTTCTGGCTCTTCTTG--GAGACGCGACCGGTGGCATCACCTC

HSVd.gv034 CCTTCTTCTTT-ACCTTCTTCTGGCTCTTCTTG--GAGACGCGACCGGTGGCATCACCTC

HSVd.gv030 CCTTCTTCTTTTACCTTCTTCTGGCTCTTCCGAT-GAGACGCGACCGGTGGCACCCCTGC

HSVd.gv033 CCTTCTTCTTT-ACCTTCTTCTGGCTCTTCTTG--GAGACGCGACCGGTGGCACCCCTGC

HSVd.gv057 CCTTCTTCTTT-ACCTTCTTCTGGCTCTTCCGATGGAGACGCGACCGGTGGCATCACCTC

HSVd.gv038 CCTTCTTCTTT-ACCTTCTTCTGGCTCTTCTTG--GAGACGCGACCGGTGGCACCCCTGC

HSVd.gv050 C-TTCTTCTTT-ACCTTCTTCTGGCTCTTCCGATGGAGACGCGACCGGTGGCATCACCTC

* ********* ******** ******** ****** ******* ** * * *

**Nucleotide position** **204-207 226, 228, 230**

HSVd-g5BB TCGGTTCG-TCCCAACCTGCTTTTGTTCTATCTGAGCCTCTGCCGCGGATCCTCTCTTGA

HSVd.gv003 TCGGTTCG-TCCCAACCTGCTTTTGTTCTATCTGAGCCTCTGCCGCGGATCCTCTCTTGA

HSVd-gRiesling TCGGTTCG-TCCCAACCTGCTTTTGTTCTATCTGAGCCTCTGCCGCGGATCCTCTCTTGA

HSVd.gv009 TCGGTTCG-TCCCAACCTGCTTTTGTTCTATCTGAGCCTCTGCCGCGGATCCTCTCTTGA

HSVd.gv002 TCGGTTCG-TCCCAACCTGCTTTTGTTCTATCTGAGCCTCTGCCGCGGATCCTCTCTTGA

HSVd-grape TCGGTTCG-TCCCAACCTGCTTTTTGTCTATCTGAGCCTCTGCCGCGGATCCTCTCTTGA

HSVd.gv061 TCGGTTCG-TCCCAACCTGCTTTTTGTCTATCTGAGCCTCTGCCGCGGATCCTCTCTTGA

HSVd.gv042 TCGGTTCG-TCCCAACCTGCTTTTTGTCTATCTGAGCCTCTGCCGCGGATCCTCTCTTGA

HSVd.gv051 TCGGTTCG-TCCCAACCTGCTTTTTGTCTATCTGAGCCTCTGCCGCGGATCCTCTCTTGA

HSVd.gv059 TCGGTTCG-TCCCAACCTGCTTTTTGTCTATCTGAGCCTCTGCCGCGGATCCTCTCTTGA

HSVd.gv039 TCGGTTCG-TCCCAACCTGCTTTTTGTCTATCTGAGCCCCTGCCGCGGATCCTCTCTTGA

HSVd.gv047 TCGGTTCG-TCCCAACCTGCTTTTTGTCTATCTGAGCCTCTGCCGCGGATCCTCTCTTGA

HSVd.gv056 TCGGTTCG-TCCCAACCTGCTTTTTGTCTATCTGAGCCTCTGCCGCGGATCCTCTCTTGA

HSVd.gv058 TCGGTTCG-TCCCAACCTGCTTTTTGTCTATCTGAGCCTCTGCCGCGGATCCTCTCTTGA

HSVd.gv001 TCGGTTCG-TCCCAACCTGCTTTTTGTCTATCTGAGCCTCTGCCGCGGATCCTCTCTTGA

HSVd.gv007 TCGGTTCG-TCCCAACCTGCTTTTTGTCTATCTGAGCCTCTGCCGCGGATCCTCTCTTGA

HSVd.gv035 TCGGTTCG-TCCCAACCTGCTTTTTGTCTATCTGAGCCTCTGCCGCGGATCCTCTCTTGA

HSVd.gv045 TCGGTTCG-TCCCAACCTGCTTTTTGTCTATCTGAGCCTCTGCCGCGGATCCTCTCTTGA

HSVd.gv054 TCGGTTCG-TCCCAACCTGCTTTTTGTCTATCTGAGCCTCTGCCGCGGATCCTCTCTTGA

HSVd.gv008 TCGGTTCG-TCCCAACCTGCTTTTTGTCTATCTGAGCCTTTGCCGCGGATCCTCTCTTGA

HSVd.gv040 TCGGTTCG-TCCCAACCTGCTTTTTGTCTATCTGAGCCTCTGCCGCGGATCCTCTCTTGA

HSVd.gv052 TCGGTTCG-TCCCAACCTGCTTTTTGTCTATCTGAGCCTCTGCCGCGGATCCTCTCTTGA

HSVd.gv036 TCGGTTCG-TCCCAACCTGCTTTTTGTCTATCTGAGCCTCTGCCGGGGATCCTCTCTTGA

HSVd.gv055 TCGGTTCG-TCCCAACCTGCTTTTTGTCTATCCGAGCCTCTGCCGCGGAACCTCTCTTGA

HSVd.gv005 TCGGTTCG-TCCCAACCTGCTTT--GTCTATCTGAGCCTCTGCCGCGGATCCTCTCTTGA

HSVd.gv037 TCGGTTCG-TCCCAACCTGCTTTTTGTCTATCTGAGCCTCTGCCGCGGATCCTCTCTTGA

HSVd.gv027 TCGGTTCG-TCCCAACCTGCTTTTTGTCTATCTGAGCCTCTGCCGCGGATCCTCTCTTGA

HSVd.gv032 TCGGTTCG-TCCCAACCTGCTTTTTGTCTATCTGAGCCTCTGCCGCGGATCCTCTCTTGA

HSVd.gv043 TCGGTTCG-TCCCAACCTGCTTTTTGTCTATCTGAGCCTCTGCCGCGGATCCTCTCTTGA

HSVd.gv053 TCGGTTCG-TCCCAACCTGCTTTTTGTCTATCTGAGCCTCTGCCGCGGATCCTCTCTTGA

HSVd.gv062 TCGGTTCG-TCCCAACCTGCTTTTTGTCTATCTGAGCCTCTGCCGCGGATCCTCTCTTGA

HSVd.gv044 TCGGTTCG-TCCCAACCTGCTTTTTGTCTATCTGAGCCTCTGCCGCGGATCCTCTCTTGA

HSVd.gv049 TCGGTTCG-TCCCAACCTGCTTTTTGTCTATCTGAACCTCTGCCGCGGATCCTCTCTTGA

HSVd.gv004 TCGGTTCG-TCCCAACCTGCTTTTTGTCTATCTGAGCCTCTGCCGCGGATCCTCTCTTGA

HSVd.gv006 TCGGTTCG-TCCCAACCTGCTTTTTGTCTATCTGAGCCTCTGCCGCGGATCCTCTCTTGA

HSVd.gv046 TCGGTTCG-TCCCAACCTGCTTTTTGTCTATCTGAGCCTCTGCCGCGGATCCTCTCTTGA

HSVd.gv011 TCGGTTCG-TCCCAACCTGCTTTTTGTCTGTCTGAGCCTCTGCCGCGGATCCTCTCTTGA

HSVd.gv060 TCGGTTCG-TCCCAACCTGCTTTTTGTCTATCTGAGCCTCTGCCGCGGATCCTCTCTTGA

HSVd.gv048 TCGGTTCG-TCCCAACCTGCTTTTTGTCTATCTGAGCCTCTGCCGCGGATCCTCTCTTGA

HSVd.gv041 TCGGTTCG-TCCCAACCTGCTTTTTGTCTATCTGAGCCTCTGCCGCGGATCCTCACTTGA

HSVd.gv029 TCGGTTCG-TCCCAACCTGCTTTTTGTCTATCTGAGCCTCTGCCGCGGATCCTCTCTTGA

HSVd.gv031 TCGGTTCG-TCCCAACCTGCTTTTTGTCTATCTGAGCCTCTGCCGCGGATCCTCTCTTGA

HSVd.gv034 TCGGTTCG-TCCCAACCTGCTTTTTGTCTATCTGAGCCTCTGCCGCGGATCCTCTCTTGA

HSVd.gv030 TCGGTTCG-TCCCAACCTGCTTTTTGTCTATCTGAGCCTCTGCCGCGGATCCTCTCTTGA

HSVd.gv033 TCGGTTCG-TCCCAACCTGCTTTTTGTCTATCTGAGCCTCTGCCGCGGATCCTCTCTTGA

HSVd.gv057 TCGGTTCGCTCCCAACCTGCTTTTGTTCTATCTGAGCCTCTGCCGCGGATCCTCTCTTGA

HSVd.gv038 TCGGTTCGCTCC-AACCTGCTTTTGTTCTATCTGAGCCTCTGCCGCGGATCCTCTCTTGA

HSVd.gv050 TCGGTTCGCTCCCAACCTGCTTTTGTTCTATCTGAGCCTCTGCCGCGGATCCTCTCTTGA

******** *** ********** *** ** ** ** ***** *** **** *****

**Nucleotide position 240/241 256-257**

HSVd-g5BB GCCCCT

HSVd.gv003 GCCCCT

HSVd-gRiesling GCCCCT

HSVd.gv009 GCCCCT

HSVd.gv002 GCCCCT

HSVd-grape GCCCCT

HSVd.gv061 GCCCCT

HSVd.gv042 GCCCCT

HSVd.gv051 GCCCCT

HSVd.gv059 GCCCCT

HSVd.gv039 GCCCCT

HSVd.gv047 GCCCCT

HSVd.gv056 GCCCCT

HSVd.gv058 GCCCCT

HSVd.gv001 GCCCCT

HSVd.gv007 GCCCCT

HSVd.gv035 GCCCCT

HSVd.gv045 GCCCCT

HSVd.gv054 GCCCCT

HSVd.gv008 GCCCCT

HSVd.gv040 GCCCCT

HSVd.gv052 GCCCCT

HSVd.gv036 GCCCCT

HSVd.gv055 GCCCCT

HSVd.gv005 GCCCCT

HSVd.gv037 GCCCCT

HSVd.gv027 GCCCCT

HSVd.gv032 GCCCCT

HSVd.gv043 GCCCCT

HSVd.gv053 GCCCCT

HSVd.gv062 GCCCCT

HSVd.gv044 GCCCCT

HSVd.gv049 GCCCCT

HSVd.gv004 GCCCCT

HSVd.gv006 GCCCCT

HSVd.gv046 GCCCCT

HSVd.gv011 GCCCCT

HSVd.gv060 GCCCCT

HSVd.gv048 GCCCCT

HSVd.gv041 GCCCCT

HSVd.gv029 GCCCCT

HSVd.gv031 GCCCCT

HSVd.gv034 GCCCCT

HSVd.gv030 GCCCCT

HSVd.gv033 GCCCCT

HSVd.gv057 GCCCCT

HSVd.gv038 GCCCCT

HSVd.gv050 GCCCCT

******

-----

* Nucleotide with yellow background showed major mutation positions. Those with red background showed singleton mutations which we did not consider as major or minor mutation position in the text.
